# The associations of socioeconomic status with incident dementia and Alzheimer’s disease are modified by leucocyte telomere length: a population-based cohort study

**DOI:** 10.1038/s41598-023-32974-x

**Published:** 2023-04-15

**Authors:** Ka Yan Lai, Chris Webster, Sarika Kumari, John E. J. Gallacher, Chinmoy Sarkar

**Affiliations:** 1 Healthy High Density Cities Lab, HKUrbanLab, The University of Hong Kong, Knowles Building, Pokfulam Road, Hong Kong Special Administrative Region China; 2 Department of Urban Planning and Design, The University of Hong Kong, Knowles Building, Pokfulam Road, Hong Kong Special Administrative Region China; 3 Dementias Platform UK, Department of Psychiatry, Warneford Hospital, University of Oxford, Oxford, OX3 7JX UK

**Keywords:** Biomarkers, Risk factors, Dementia, Socioeconomic scenarios, Epidemiology

## Abstract

Socio-economic status (SES) and biological aging are risk factors for dementia, including Alzheimer’s disease, however, it is less clear if the associations with SES vary sufficiently across different biological age strata. We used data from 331,066 UK Biobank participants aged 38–73 with mean follow-up of 12 years to examine if associations between SES (assessed by educational attainment, employment status and household income) and dementia and Alzheimer’s disease are modified by biological age (assessed by leucocyte telomere length: LTL). Diagnosis of events was ascertained through hospital admissions data. Cox regressions were used to estimate hazard ratios [HRs]. A consistent dose–response relationship was found, with participants in low SES and shorter LTL strata (double-exposed group) reporting 3.28 (95% confidence interval [CI] 2.57–4.20) and 3.44 (95% CI 2.35–5.04) times higher risks of incident dementia and Alzheimer’s disease respectively, compared to those of high SES and longer LTL (least-exposed group). Of interest is a synergistic interaction between SES and LTL to increase risk of dementia (RERI 0.57, 95% CI 0.07–1.06) and Alzheimer’s disease (RERI 0.79, 95% CI 0.02–1.56). Our findings that SES and biological age (LTL) are synergistic risk factors of dementia and Alzheimer’s disease may suggest the need to target interventions among vulnerable sub-groups.

## Introduction

Dementia is a syndrome with multiple pathologies characterized by progressive neurodegeneration leading to cognitive and functional decline. It is estimated to affect 57 million people globally^1^. For the UK, estimated social and healthcare costs are currently around £35 billion, rising to £80.1 billion by 2040^2,3^. It is also estimated that 35% of population dementia risk is attributable to modifiable social and environmental risk factors, including socioeconomic status (SES)^4^.

The role of SES in elevating dementia risk is well documented^5^. Epidemiologic studies have shown that higher education attainment (as a cognitive-stimulating activity)^6,7^ and household income (permitting more opportunities for healthy lifestyle)^8,9^ are protective for dementia. Conversely, low occupational employment was found to be associated with increased dementia risk^10^. Less clear, however, are the biological mechanisms underlying these associations.

Biological aging is the progressive loss of physiological function relative to chronological age, and captures the potential progressive impairment of metabolic and physiologic capabilities of human body at cellular and molecular levels^11^. Telomere length (TL) has been suggested as an indicator of biological aging. Telomeres are thousands of repeated TTAGGG hexa-nucleotide sequences that cap the ends of chromosomes and are responsible for chromosome stability. Being located at the tip of chromosomes, telomeres are not fully replicated during DNA replication leading to an attrition in length accompanying each successive cell division. Variation in TL between individuals can be used as a marker of biological aging^12^. Attrition in length also stems from the effects of life stressors^13^, inflammation, DNA damage, ageing, and lifestyle factors such as smoking^14^. Specifically, socioeconomic disadvantage constitutes an important marker of psychosocial stress and several studies have found evidence of associations between socioeconomic disadvantage and telomere shortening^15,16^. Evidence from the National Health and Nutrition Examination Survey (NHANES) study comprising n = 5,360 participants aged 20–84 years showed that participants completing high school education had relatively shorter telomeres than college graduates^17^. Consistent results were obtained from a sample of n = 416 participants aged 53–76 years from the Whitehall II epidemiological cohort, with lower educational attainment being associated with shorter leucocyte telomere length (LTL)^18^. In a study of n = 1,552 female twins aged 18–75 years, individuals belonging to lower SES category was found to have shorter telomeres relative to peers in higher SES, with the white blood cell telomeres in the lower SES category being shorter on average by 140.3 base pairs after accounting for other risk factors such as body size, smoking and physical activity^19^. A study of n = 1,026 found that higher parental SES was associated with 1.8% longer TL among newborn boys^20^. Another cross sectional study of n = 341 mixed race participants found that younger adults with higher burden of discrimination (race and sex) had shorter TL, while similar associations were reported among female from higher SES strata^21^. A recent longitudinal study of n = 1,031 participants from the Multi-Ethnic Study of Atherosclerosis (MESA) reported that improvements in neighbourhood-level SES were protective on the rate of telomere attrition^22^.

Telomere length (TL) has also been shown to be directly associated with risk of dementia and Alzheimer’s disease (see Supplementary Table S1 for study details). Briefly, one prospective study comprising n = 1,973 subjects aged ≥ 65 years from New York city found each kilobase pair attrition in TL was associated with 21% higher risk of dementia^23^. Consistently, longer TL was found to be protective on dementia risk among stoke survivors, aged ≥ 75 years in the UK^24^. A systematic review of 13 studies found consistent evidence of shorter TL among 860 Alzheimer’s disease patients relative to 2,022 controls with standardized mean difference of − 0.984 (95% CI −1.433 to −0.535)^25^. A study from Adelaide, Australia reported shorter TL in buccal and white blood cells in clinically diagnosed Alzheimer’s patients relative to healthy age-matched controls^26^. Recently, a two-sample Mendelian randomization study leveraging on summary statistics data extracted from largescale GWAS inferred that longer leucocyte TL have a protective effect on risk of Alzheimer’s disease^27^. Nonetheless, one large prospective study comprising n = 1,961 Dutch participants reported an U-shaped association, with higher risks of Alzheimer’s disease in the lowest and highest tertiles of TL relative to the middle tertile^28^. While, another cross sectional study comprising n = 2,210 participants demonstrating exceptional longevity from USA and Denmark reported null associations between leucocyte TL and cognitive performance in both cognitively unimpaired and demented sub-groups^29^.

Although independent positive associations of lower SES and shorter TL with risk of dementia have been established in several previous studies, there has thus far been no study examining synergistic interactions to inform targeted interventions for vulnerable subgroups. Here we aim to investigate how SES and TL interact to affect dementia risk. Using large-scale data from UK Biobank, we examine the effect-modification of biological age as measured by LTL on the associations of a composite measure of SES comprising highest educational attainment, employment status, and household income with incident dementia and Alzheimer’s disease.

## Methods

### Study population

This prospective study included participants from the UK Biobank, a nationwide population-based cohort of over 500,000 adults aged 37–73 years at baseline^30^. Participants were recruited between April 2006 and December 2010 by postal invitation from the National Health Service patient registry (response rate of 5.5%) within approximately 25 miles of 22 assessment centres across England, Scotland and Wales. Participants completed touchscreen questionnaires, underwent nurse-administered face-to-face interviews, had anthropometric assessments, and provided biological samples (blood, saliva and urine). Detailed protocol of the UK Biobank are available online^31^.

## Study variables

### Ascertainment of clinically diagnosed dementia and Alzheimer’s disease

Date and cause of hospital admissions were derived from Hospital Episode Statistics Admitted Patient Care (England), Patient Episode Database (Wales), and General/ Acute Inpatient and Day Case—Scottish Morbidity Record (Scotland). Incident clinically diagnosed all-cause dementia diagnosis was defined as primary or secondary diagnosis of dementia from the International Classification of Diseases—10th Revision (ICD 10), coded as F00 (Dementia in Alzheimer disease), F01 (vascular dementia), F02 (dementia in other diseases), F03 (unspecified dementia), G30 (Alzheimer disease), G31 (other dementias) or A81 (Sporadic Creutzfeld Jacob disease). Incident Alzheimer’s disease diagnosis was defined as ICD10 code F00 (Dementia in Alzheimer disease) or G30 (Alzheimer Disease) (see *Appendix* Table S3). Follow-up was censored at the date of hospital admission, date of all-cause death (from death certificates), or study end date (31 March 2021), whichever occurred earlier.

### Measurement of composite socioeconomic status

Individual-level SES, our primary exposure was derived from a latent class model based on three socioeconomic factors; highest educational attainment, employment status and household income in accordance with a prior study^32^. Latent class analysis (LCA) is a well-validated data-driven technique that employs observed categorical domain variables to identify unobserved latent variable with mutually exclusive latent classes^33^. The three socioeconomic factors were collected from cohort participants based on structured self-reported questionnaires. Highest educational attainment was included as a seven-category variable based on the International Standard Classification of Education in the latent class model and categorized as college or university degree, A levels/AS levels, O levels/GCSEs, CSEs, NVQ or HND or HNC, other professional qualifications (for example nursing, teaching), and none of the above equivalent to less than high school. Employment status was a three-category variable coded as employed or self-employed, retired, and others (which included looking after home, being unable to work due to sickness or disability, unemployed, doing unpaid or voluntary work or student). Average total household income before tax was divided into five groups (< £18,000, £18,000 to 30,999, £31,000 to 51,999, £52,000 to 100,000, and > £100,000). Three SES classes (low, medium and high) were subsequently identified based on item-response probabilities using *gsem* command in Stata^34^. Maximum likelihood method, which permits the use of all data available including those with missingness, was employed in the LCA models. Iterations were undertaken before the final model was selected. Model selection was determined by using Bayesian information criteria (BIC) and Akaike information criterion (AIC). The convergence and performance of the latent class models were checked for two, three and four or more classes^32^. In our iterations, models with two and three classes performed the best. The AIC and BIC values for the latent class model with two classes was reported as 3.78 × 10^6^ (both statistic), while that for the model with three classes are 3.76 × 10^6^. Based on these fit statistic, we selected the latent class model with three classes to define individual-level SES for this study. The mean posterior probability values of the latent class model with three classes are 0.73, 0.81 and 0.84 for classes 1, 2 and 3 respectively (Supplementary Methods S1 and Table S2).

### Measurement of biological age

Leucocyte telomere length (LTL), expressed as the ratio of telomere repeat copy number to the single copy gene (T/S), was extracted from peripheral blood leucocytes of the UK Biobank participants and measured via validated qPCR methods^35^. LTL was adjusted for technical parameters (including enzyme, PCR machine, primer, operator, temperature, humidity, hours from 6 a.m., pipetting robot and extraction method), log_e_ transformed and Z-standardized. We developed a composite measure of biological age which accounted for the potential correlations between chronological age and LTL as well as the combined effects of inflammation and chronic diseases. As in previous study^36^, biological age was defined as residuals of LTL additionally adjusting for chronological age, serum C-reactive protein (from immunoturbidimetric high-sensitivity analysis) and any of the 46 curated chronic comorbidities (Supplementary Table S4) comprising cardiovascular diseases, cancer, diabetes, hypertension, psychiatric disorders and respiratory diseases. Serum CRP value expressed as mg/L was measured by immunoturbidimetric high-sensitivity analysis with the use of a Beckman Coulter AU5800^37^.

### Covariates

Following a socio-ecological perspective, model covariates were identified a priori, informed from published literature^4^. Demographic covariates included age at baseline, sex and ethnicity. A composite healthy lifestyle score was developed based on smoking status, alcohol intake frequency and dietary factors assessed at baseline as in previous literature^38–40^. A score of 1 was assigned to participants who achieved the healthy standard in each lifestyle component (non-current smoker; alcohol not consumed daily/almost daily; consumed ≥ 4 of 7 types of food following dietary recommendations as previously). The healthy score ranged between 0 and 3 was subsequently categorized as favorable (a score of 3), intermediate (a score of 2) and unfavorable (a score of 0 or 1) following the data distribution. The number of leisure and social activities per week was coded as none, one or more. Hearing difficulty was coded as self-reported deaf or having difficulty with hearing (coded as 1), and without hearing difficulty (coded as 0). Abdominal obesity was defined in terms of waist-hip ratio (WHR) ≥ 0.85 cm for women and ≥ 0.90 cm for men. Frailty was assessed at baseline from five phenotypes; weight loss, exhaustion, low grip strength, low physical activity and slow gait speed and coded as non-frail, frail/pre-frail^41,42^. Among the environmental variables, urbanicity was a composite measure of densities of housing, retail and public transport, and urban centrality around the participants’ residence (coded as quintiles 1, 2–4 and 5) as previously^43,44^. Neighbourhood deprivation (coded as quintiles 1, 2–4 and 5) was measured by the Townsend deprivation index (Supplementary Methods S2 for additional details).

### Statistical analyses

Descriptive characteristics across the three SES categories are presented as mean and standard deviation for continuous variables, and frequency and percentage for categorical variables. We employed Nelson-Aalen hazard method to calculate the cumulative hazard for incident dementia based on SES categories (3 groups), and subgroups by SES and LTL strata (9 groups) over time.

Cox proportional hazard regression models estimating hazard ratios (HR) and 95% confidence interval (CI) for associations of SES and TL with risks of incident dementia and Alzheimer’s disease were developed. Person-time in months were calculated from the date of entry into the cohort until the date of hospitalization or date of death, or the study end date, whichever occurred earlier. The proportional hazards assumption was tested by using the Schoenfeld residual test. We excluded events prior to baseline assessment. To minimize chance of reverse causality, we performed landmark analyses by excluding events within 3 years of entry in to the study.

We examined independent associations of SES and LTL with incident dementia and Alzheimer’s disease diagnosis. HRs in the medium and low SES categories were reported in reference to the high SES category. Linear test was conducted to estimate the associational trend across the three exposure categories. To examine if biological age moderated the associations, we conducted stratified analyses by LTL. LTL was coded as a three-level categorical variable based on cut-offs at the 25th and 75th percentiles, with participants at the top 25th percentile with longer LTL taken as the reference group. Lastly, we examined joint associations between SES and LTL on incident events by stratifying participants into 9 groups by SES (low, medium and high) and LTL (long medium, short), with participants in the high SES and long LTL strata acting as the reference group. Biologic interaction on an additive scale was expressed as relative excess risk due to interaction (RERI) with the corresponding 95% CIs obtained by estimating the separate and joint effects of SES and LTL on incident dementia and Alzheimer’s disease. In the RERI calculations, we defined participants with low SES and short LTL as the double-exposed group, while those with high SES and long LTL strata as the reference category^45,46^. The models included age and sex, ethnicity, healthy lifestyle score, social activities, hearing difficulty, clinical phenotypes (abdominal obesity and frailty index), urbanicity, neighbourhood deprivation and SES/LTL. To fulfill the proportionate hazards assumption, sex was inputted using the strata option throughout the analysis.

We performed several sensitivity tests. First, we repeated our models with single socioeconomic factors (highest education attainment, employment status and household income) to examine their independent associations with incident dementia and Alzheimer’s disease. Second, we repeated our primary analyses using combined cases of both incident dementia diagnosis and mortality. Data on dementia mortality was extracted from death register, with the date of death obtained from death certificates held by the National Health Service (NHS) Information Centre (England and Wales) and the NHS Central Register in Scotland. Third, we reran the LCA model comprising the three individual socioeconomic factors as well as the Townsend deprivation index to additionally take into account the effect of neighbourhood deprivation within the composite SES measure. Fourth, we further reran analyses with adjustment for number of household members (coded as living alone, two, three, and more than three) to take into account variability in household size. Fifth, we reran models after additionally adjusting for comorbidities in the main models as covariates to examine robustness of our findings (with comorbidities being removed from the residual analysis for LTL). Sixth, for the employed subpopulation, we additionally reran models examining associations between occupation categories and incident dementia and Alzheimer’s disease. Occupation categories were coded as a five-factor variable comprising other occupations, managers and senior officials, professional occupations, associate professional and technical occupations, and administrative and secretariat occupations. Other occupations included manual occupations such as skilled trades occupations/personal service occupations/sales and customer service occupations/process, plant and machine operatives/elementary occupations, and was taken as a reference category. Seventh, we examined potential modifying effect of age by conducting stratified analyses by age groups (< 65 versus ≥ 65 years). Lastly, we performed multiple imputation by chained equations with 20 imputation sets^47^ to account for missing data across covariates on a sample of 422,907 participants with complete data on outcome and exposure variables. All covariates, exposure and outcome variables in the fully-adjusted model and the follow-up time were used to impute missingness.

Data were analyzed from November 2021 to August 2022. All analyses were performed in Stata software version 16.1.

### Ethics approval and consent to participants

Ethical approval of UK Biobank was obtained from the National Health Service National Research Ethics Service (Ref: 11/NW/0382) and subsequently renewed (Ref: 16/NW/0274 and 21/NW/0157). We also obtained institutional ethical approval from The University of Hong Kong’s Human Research Ethics Committee (Refs: EA220302 and EA1904019). Informed electronic consent was acquired from all cohort participants when they visited the assessment centres.

## Results

From the UK Biobank population sample of 502,650 individuals, participants were omitted from the analysis due to withdrawal (n = 191), diagnosed with dementia during the first three years of follow-up (the landmark period) or at baseline (n = 377), and missingness on one or more SES exposure (n = 79,175) or covariates (n = 91,841). The remaining 331,066 participants were used in a complete sample analysis (Supplementary Figure S1). There was no systematic difference between analytic sample and excluded sample (Supplementary Table S5). Over 3,989,206 person-years of follow-up (mean follow-up period = 12 years; IQR: 11.5–13.0 years), 3,313 incident cases of dementia were recorded, of which 1,423 were of Alzheimer’s disease.

Mean age of the sample at baseline was 56 (SD = 8), with 52% women (Table 1). The LCA model for composite SES categorized participants into three SES groups for high (n = 111,525, 34%), medium (n = 138,373, 42%) and low (n = 81,168, 25%) socioeconomic status. In general, participants with dementia belonged to the older age sub-group and with educational qualification less than high school, lower household income, obese and with lower grip strength, gait speed, physical activity levels, shorter leucocyte telomere length (LTL) and more comorbidities. The mean LTL of analytic sample was 0.01 (SD = 1.00, range = − 15.28–12.14). As expected older participants belonging to low SES strata had shorter LTL. The mean LTL among participants aged < 65 years and of low, medium and high SES strata were − 0.10 (SD = 1.00), 0.04 (SD = 0.99) and 0.15 (SD = 0.99) respectively. While, the mean LTL in sub-group aged ≥ 65 years and belonging to low, medium and high SES strata were − 0.30 (SD = 0.98), − 0.27 (SD = 0.98) and − 0.25 (SD = 0.97) respectively.

**Table 1:** Baseline characteristics of UK Biobank target sample (N = 331,066) according to dementia status.

Characteristics	All	Without dementia	With dementia
	(n = 331,066)	(n = 327,753)	(n = 3,313)
Socio-demographics and lifestyle factors			
Men	158,460 (48)	156,534 (48)	1,926 (58)
Mean (SD) age (years)	56.14 (8.08)	56.06 (8.07)	64.19 (4.63)
Ethnicity			
White	317,813 (96)	314,601 (96)	3,212 (97)
Non-white	13,253 (4)	13,152 (4)	101 (3)
Highest educational qualification			
Other professional	16,891 (5)	16,672 (5)	219 (7)
NVQ/HND/HNC	21,908 (7)	21,643 (7)	265 (8)
CSEs	17,649 (5)	17,580 (5)	69 (2)
O levels/GCSEs	71,338 (22)	70,698 (22)	640 (19)
A levels/AS levels	39,036 (12)	38,703 (12)	333 (10)
College or University degree	117,357 (35)	116,571 (36)	786 (24)
Less than high school	46,887 (14)	45,886 (14)	1,001 (30)
Employment status			
Employed or self-employed	190,851 (58)	190,121 (58)	730 (22)
Retired	116,582 (35)	114,216 (35)	2,366 (71)
Others	23,633 (7)	23,416 (7)	217 (7)
Household income			
< £18,000	70,300 (21)	68,825 (21)	1,475 (45)
£18,000–£30,999	83,761 (25)	82,712 (25)	1,049 (32)
£31,000–£51,999	88,216 (27)	87,704 (27)	512 (15)
£52,000–£100,000	70,126 (21)	69,896 (21)	230 (7)
≥ £100,000	18,663 (6)	18,616 (6)	47 (1)
Healthy diet	147,260 (44)	145,752 (44)	1,508 (46)
Not frequent alcohol consumer	259,604 (78)	257,058 (78)	2,546 (77)
Not current smoker	297,202 (90)	294,253 (90)	2,949 (89)
Healthy lifestyle score			
0–1	62,371 (19)	61,709 (19)	662 (20)
2	157,680 (48)	156,148 (48)	1,532 (46)
3	111,015 (34)	109,896 (34)	1,119 (34)
Social activities			
None	97,533 (29)	96,519 (29)	1,014 (31)
One or more	233,533 (71)	231,234 (71)	2,299 (69)
Clinical phenotypes			
Abdominal obesity^a^			
Obese	162,259 (49)	160,177 (49)	2,082 (63)
Mean (SD) waist circumference (cm)	90.27 (13.34)	90.23 (13.34)	93.62 (13.43)
Mean (SD) Hip circumference (cm)	103.31 (9.03)	103.31 (9.03)	103.51 (9.18)
Frailty			
Frail/pre-frail	131,250 (40)	129,440 (39)	1,810 (55)
Weight change compared with 1 year age			
Weight-loss	50,552 (15)	49,918 (15)	634 (19)
Grip strength			
Low grip strength	42,408 (13)	41,552 (13)	856 (26)
Gait speed			
Low gait speed	22,920 (7)	22,332 (7)	588 (18)
Exhaustion			
More than half the days/nearly everyday	38,490 (12)	38,014 (12)	476 (14)
Physical activities			
Low physical activity	26,081 (8)	25,690 (8)	391 (12)
Hearing difficulty			
Deaf or having difficulty with hearing	84,430 (26)	83,229 (25)	1,201 (36)
Mean (SD) Leucocyte telomere length^b^	0.01 (1.00)	0.01 (1.00)	− 0.28 (0.98)
Mean (SD) C-reactive protein (mg/L)	2.51 (4.23)	2.51 (4.22)	2.79 (4.64)
Comorbidities at baseline			
Cancer	19,756 (6)	19,497 (6)	259 (8)
Cardiovascular disease	46,703 (14)	45,733 (14)	970 (29)
Diabetes	15,928 (5)	15,448 (5)	480 (14)
Hypertension	94,283 (28)	92,655 (28)	1,628 (49)
Psychiatric disorder	38,738 (12)	38,303 (12)	435 (13)
Respiratory disease	71,676 (22)	70,857 (22)	819 (25)
Neighbourhood environment			
Urbanicity			
Tertile 1 (least urbanized)	68,666 (21)	68,107 (21)	559 (17)
Tertile 2–4	197,630 (60)	195,536 (60)	2,094 (63)
Tertile 5 (most urbanized)	64,770 (20)	64,110 (20)	660 (20)
Townsend deprivation index			
Tertile 1 (least deprived)	69,809 (21)	69,142 (21)	667 (20)
Tertiles 2–4	203,052 (61)	201,127 (61)	1,925 (58)
Tertile 5 (most deprived)	58,205 (18)	57,484 (18)	721 (22)

Values are numbers (percentage) unless stated otherwise.

^a^Obese is defined as waist–hip ratio (WHR) ≥ 0.90 cm for men or ≥ 0.85 cm for women. Non-obese is defined as WHR < 0.90 cm for men or < 0.85 cm for women.

^b^Leucocyte telomere length was Z-standardised, log transformed and adjusted for technical parameters.

### Cumulative hazards of incident dementia and Alzheimer’s disease across SES and LTL strata

**Figure 1:**
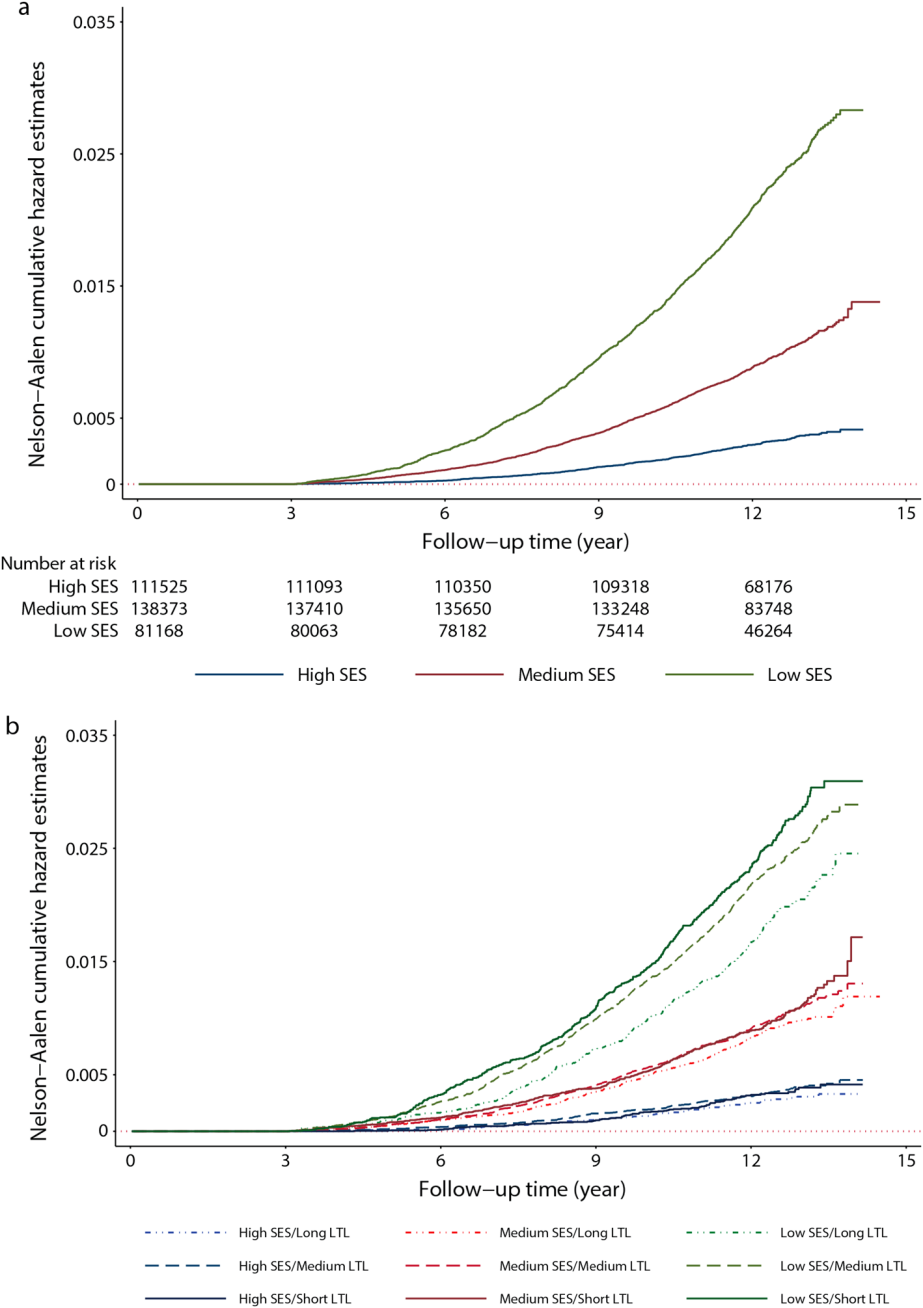
Nelson-Aalen cumulative hazard estimates showing cumulative risk of incident dementia. Analyses were performed based on data of 331,066 participants and stratified by: (**a**) socioeconomic status (SES) and (**b**), SES and leucocyte telomere length, LTL (residual of LTL, adjusted for chronological age, C-reactive protein and co-morbidities was employed as marker of biological age). The plotted lines represent the cumulative risk of incident dementia using Nelson-Aalen cumulative hazard estimates.

Nelson-Aalen hazard estimates showed consistently higher cumulative risks of incident dementia for lower SES strata (Fig. 1a). The cumulative risks for incident dementia at 12 years were 2.1% (95% CI 1.99–2.20) for low SES, 0.88% (95% CI 0.83–0.94) for medium and 0.30% (95% CI 0.27–0.33) for the high SES stratum. Further stratification by SES and LTL revealed a cumulative risk for incident dementia of 2.33% (95% CI 2.12–2.56) in participants of low SES and short LTL, while it was only 1.67% (95% CI 1.49–1.87) for the low SES and long LTL category (Fig. 1b). A similar pattern was observed for Alzheimer’s disease with the cumulative risks at 12 years being 0.94% (95% CI 0.87–1.01) for low SES, 0.37% (95% CI 0.34–0.40) for medium and 0.12% (95% CI 0.10–0.14) for the high SES stratum.

### Associations of individual-level socioeconomic status (SES) with incident dementia and Alzheimer’s disease

Multivariate analysis found strong associations of dementia and Alzheimer’s disease with SES (Table 2). Relative to high SES, after adjustment for age and including sex as strata, the hazard ratio (HR) for incident dementia increased with lower SES, going from HR 1.66 (95% CI 1.47–1.88) for medium SES to HR 3.02 (95% CI 2.68–3.40) for low SES strata (*p* trend < 0.001) (model 1). Further adjustment for ethnicity, lifestyle, social activities, hearing difficulty, obesity, frailty, urbanicity and neighbourhood deprivation moderated these associations slightly with HR 1.60 (95% CI 1.42–1.80) for medium SES and HR 2.59 (95% CI 2.29–2.92) for low SES strata without making any material difference to the significance of the trend (*p* trend < 0.001) (model 2). Further adjustment for biological age (as modelled from residual of LTL) resulted in little change in the effect estimates with HR 1.59 (95% CI 1.41–1.80) for medium SES and HR 2.58 (95% CI 2.28–2.91) for low SES, indicating independence of SES and LTL exposures. Relative to long LTL, after adjustment for age, ethnicity, lifestyle, social activities, hearing difficulty, obesity, frailty, urbanicity, and neighbourhood deprivation and including sex as strata, the hazard ratio for incident dementia increased with shorter LTL, going from HR 1.17 (95% CI 1.07–1.28) for medium LTL and HR 1.20 (95% CI 1.09–1.32) for short LTL strata (*p* trend < 0.001) (Supplementary Table S6). A comparable pattern of association was found for Alzheimer’s disease.

**Table 2:** Associations of individual-level socioeconomic status (SES) with risks of incident dementia and Alzheimer’s disease among UK Biobank participants.

	Total	Individual-level SES strata^d^			*p* trend
		High	Medium	Low	
	N/Events	HR (95% CI)	HR (95% CI)	HR (95% CI)	
Incident dementia					
N/Events	331,066/3,313	111,525/350	138,373/1,280	81,168/1,683	
Model 1^a^		1–Ref	1.66 (1.47–1.88)	3.02 (2.68–3.40)	< 0.001
Model 2^b^		1–Ref	1.60 (1.42–1.80)	2.59 (2.29–2.92)	< 0.001
Model 3^c^		1–Ref	1.59 (1.41–1.80)	2.58 (2.28–2.91)	< 0.001
Incident Alzheimer’s disease					
N/Events	331,066/1,423	111,525/143	138,373/528	81,168/752	
Model 1^a^		1–Ref	1.60 (1.32–1.93)	3.06 (2.54–3.68)	< 0.001
Model 2^b^		1–Ref	1.55 (1.28–1.87)	2.70 (2.23–3.25)	< 0.001
Model 3^c^		1–Ref	1.54 (1.28–1.86)	2.68 (2.22–3.24)	< 0.001

^a^Model 1 included age and sex.

^b^Model 2 included variables in Model 1 and additionally adjusted for ethnicity, healthy lifestyle score, social activities, hearing difficulty, abdominal obesity, frailty index, urbanicity and neighbourhood deprivation.

^c^Model 3 included variables in Model 2 and additionally adjusted for biological age (measured as residual of leucocyte telomere length, LTL, adjusted for chronological age, C-reactive protein and co-morbidities).

leucocyte telomere length, LTL).

^d^Individual-level SES is derived from latent class analysis by using participants’ reported data on highest education attainment, employment status and household income.

*CI* Confidence interval; *HR* Hazard ratio; *N* Number of participants.

### Associations of individual-level socioeconomic status (SES) with incident dementia and Alzheimer’s disease stratified by biological age

To investigate the interaction effect of LTL and SES on dementia risk, dementia was regressed on SES stratified by LTL (Table 3). There was a closely similar pattern of elevated risks of dementia and Alzheimer’s disease towards low SES across the LTL stratum. Relative to participants of high SES, those of low SES had HRs for incident dementia of 2.49 (95% CI 1.91–3.24) for long LTL stratum and 2.96 (95% CI 2.32–3.77) for short LTL stratum. Similarly, participants of low SES were also associated with higher risks of incident Alzheimer’s disease with HRs of 2.27 (95% CI 1.51–3.43) for long LTL and 3.17 (95% CI 2.18–4.59) for short LTL category.

**Table 3:** Associations between individual-level socioeconomic status (SES) and risks of incident dementia and Alzheimer’s disease stratified by biological age (measured as the adjusted residual of leucocyte telomere length, LTL) among UK Biobank participants.

	Total	Individual-level SES strata^b^						*p* trend
		High		Medium		Low		
	N/Events	N/Events	HR (95% CI)	N/Events	HR (95% CI)	N/Events	HR (95% CI)	
Incident dementia^a^								
1 (top 25th—long LTL^c^)	82,518/702	28,514/75	1–Ref	33,996/287	1.73 (1.33–2.24)	20,008/340	2.49 (1.91–3.24)	< 0.001
2	165,723/1,703	56,074/189	1–Ref	69,406/657	1.50 (1.27–1.77)	40,243/857	2.43 (2.06–2.87)	< 0.001
3 (bottom 25th—short LTL)	82,825/908	26,937/86	1–Ref	34,971/336	1.68 (1.32–2.14)	20,917/486	2.96 (2.32–3.77)	< 0.001
Incident Alzheimer’s disease^a^								
1 (top 25th—long LTL)	82,518/292	28,514/31	1–Ref	33,996/117	1.60 (1.07–2.40)	20,008/144	2.27 (1.51–3.43)	< 0.001
2	165,723/736	56,074/76	1–Ref	69,406/267	1.45 (1.12–1.88)	40,243/393	2.63 (2.03–3.41)	< 0.001
3 (bottom 25^th^—short LTL)	82,825/395	26,937/36	1–Ref	34,971/144	1.69 (1.16–2.44)	20,917/215	3.17 (2.18–4.59)	< 0.001

^a^Model included age, sex, ethnicity, healthy lifestyle score, social activities, hearing difficulty, abdominal obesity, frailty index, urbanicity and neighbourhood deprivation.

^b^Individual-level SES is derived from latent class analysis by using participants’ reported data on highest education attainment, employment status and household income.

^c^Residual of LTL, adjusted for chronological age, C-reactive protein and co-morbidities was defined as biological age in the study.

*CI* Confidence interval; *HR* Hazard ratio; *N* Number of participants, *LTL* Leucocyte telomere length.

**Figure 2:**
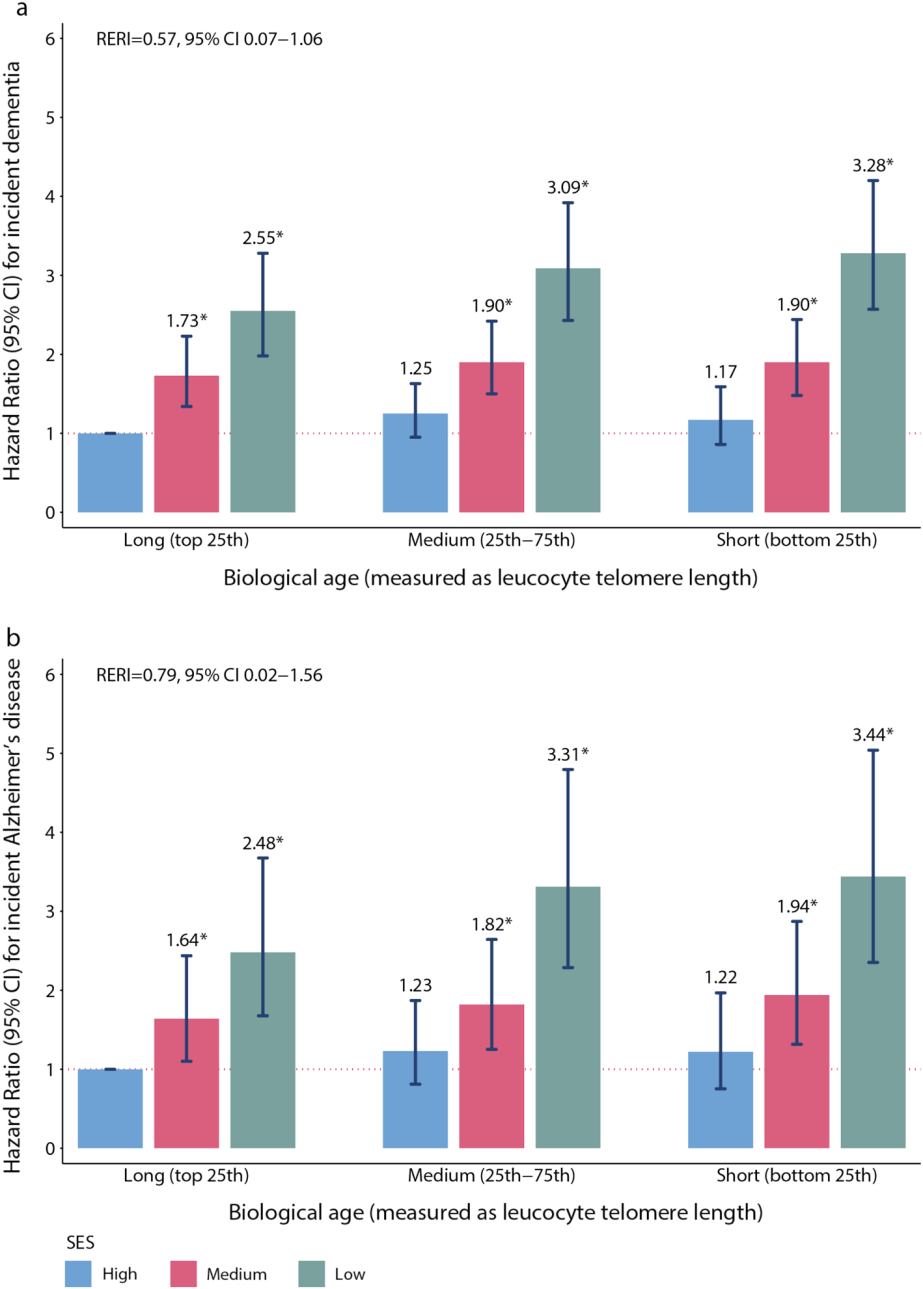
Joint associations of individual-level socio-economic status (SES) and adjusted residual leucocyte telomere length (LTL) with incident dementia and Alzheimer’s disease among UK Biobank participants. Analyses were performed based on data of 331,066 participants for (**a**) incident dementia and (**b**) incident Alzheimer’s disease. Models included age, sex, ethnicity, healthy lifestyle score, social activities, hearing difficulty, abdominal obesity, frailty index, urbanicity and neighbourhood deprivation. Residual of LTL, adjusted for chronological age, C-reactive protein and co-morbidities was defined as biological age in the study. The vertical bars indicate the hazard ratio, while the whiskers indicate the corresponding 95% confidence interval (CI). The asterisks represent statistically significant (two-sided *p* < 0.05) point estimates. Relative excess risk due to interaction (RERI) was used to examine additive interaction between SES (low SES versus high SES) and LTL (short LTL versus long LTL). See Supplementary Table S7 for detailed estimates, the corresponding 95% CI and calculations of RERI.

Joint associations of individual-level SES and LTL showed a consistent dose–response relationship for LTL and SES independently (Fig. 2). Compared with participants of high SES and longer LTL (least-exposed group), those of low SES and shorter LTL (double-exposed group) had higher HRs of 3.28 (95% CI 2.57–4.20) for incident dementia and 3.44 (95% CI 2.35–5.04) for incident Alzheimer’s disease. We found evidence of positive biologic interaction between LTL and SES, with the combined effect of the two factors in the double-exposed group exceeding the sum of their separate effects relative to the least-exposed group (Supplementary Table S7). The relative excess risk due to interaction (RERI) for both dementia (RERI = 0.57, 95% CI 0.07–1.06), and Alzheimer’s disease (RERI = 0.79, 95% CI 0.02–1.56) were positive, indicating a departure from additivity towards excess risk in double-exposed groups (i.e. synergism).

### Sensitivity analyses

Repeating analyses with single socioeconomic factors of educational attainment, household income and employment status, showed elevated risks for both incident dementia and Alzheimer’s disease among participants of lower education attainment, lower household income and those who were retired and belonging to the others category (including unemployed) (Supplementary Tables S8–10). Consistent with our primary analysis, models combining cases of incident dementia diagnosis and mortality as the outcome showed that participants of low SES and shorter LTL (double-exposed group) had higher HRs of 3.21 (95% CI 2.53–4.08) in reference to those of high SES and longer LTL (least-exposed group) with synergistic interaction (RERI = 0.62, 95% CI 0.16–1.09) (Supplementary Table S11). Similarly, the analysis with composite SES derived from LCA models comprising both multiple individual socioeconomic factors and neighborhood deprivation (measured by Townsend index of deprivation) showed consistent results as in our primary analysis, with participants of low SES and shorter LTL (double-exposed group) reporting higher HRs of 2.75 (95% CI 2.27–3.34) and 2.99 (95% CI 2.19–4.07) for incident dementia and Alzheimer’s disease respectively, in reference to their counterparts belonging to high SES and longer LTL (least-exposed group) (Supplementary Table S12). Our results also remained robust after additional adjustment for number of household members (Supplementary Table S13) and comorbidities taken as covariates (Supplementary Tables S14) with increased risk of incident dementia and Alzheimer’s disease being associated with lower SES and shorter LTL. Rerunning analyses for occupational categories showed lower risk of incident dementia and Alzheimer’s disease for participants engaged in professional occupations, and administrative and secretariat occupations relative to those who belonged to other occupational (manual) category, nonetheless the results remained insignificant (Supplementary Table S15).

Stratified analyses by age in the study subgroups showed that relative to participants aged < 65 years belonging to high SES-longest LTL tertile, those in low SES-shortest LTL tertile had higher HRs of 4.57 (95% CI 2.46–8.50) for dementia and 3.45 (95% CI 1.26–9.42) for Alzheimer’s disease. The effect sizes for age-group ≥ 65 years were more pronounced in the double-exposed category relative to least-exposed, with HRs of 6.37 (95% CI 4.84–8.38) for dementia and 7.42 (95% CI 4.82–11.44) for Alzheimer’s disease (Supplementary Tables S16, 17). Sensitivity analyses with imputed data accounting for missingness across covariates produced consistently robust results further supporting the main analyses (Supplementary Tables S18).

## Discussion

In a large prospective study, we found SES (a composite metric developed from household income, educational attainment and employment status) and leucocyte telomere length (LTL), were each independently monotonically associated with higher risks for incident dementia and Alzheimer’s disease. We also found that SES and LTL demonstrated modest interaction effects, suggesting that biological aging as measured by LTL and SES are likely to represent separate but synergistic risk pathways. Several sensitivity analyses such as rerunning models with single socioeconomic factors, combined cases of incident dementia diagnosis and mortality, LCA models based on both individual socioeconomic factors and neighborhood deprivation, and multiple imputations showed largely consistent results.

These findings are consistent with previous work. A 12-year follow-up study of 6,220 participants aged ≥ 65 from the English Longitudinal Study of Aging reported a dementia hazard ratio of 1.50 (95% CI 1.05–2.13) for lower SES^9^. Another 12-year follow-up study of 2,457 US elderly found increased hazard for incident dementia among participants with less than high school education (HR 1.47, 95% CI: 1.17–1.86)^6^. A Swedish cohort study of 931 participants aged ≥ 75 reported that low education and occupation-based SES were associated with elevated risks of dementia (RR = 2.4, 95% CI 1.5–4.0) and Alzheimer’s disease (RR = 3.1, 95% CI 1.6–5.7)^48^. For LTL, the Washington Heights study of 1,983 participants followed for 8 years reported that each kilobase pair decrease in LTL was associated with 21% increased risk for dementia^49^. However, a study of 1,961 participants followed for 8 years in Rotterdam found a U-shaped relationship between LTL and Alzheimer’s risk with HR 1.59 (95% CI 1.13–2.23) for the first tertile in reference to the middle. Of particular interest are cross-sectional findings from the HANDLS study. Among 325 individuals, interactions between LTL and SES for cognitive performance were found (b = − 2.06; *p* = 0.008) suggesting that for low SES shorter LTL is associated with poorer cognitive performance^50^. Our study extends this evidence by providing longitudinal data, at-scale, for clinically relevant outcomes.

Potential mechanisms for these associations are complex and remain an active area of research. Lower SES is associated with poorer cognitive reserve, higher levels of psychosocial stress, poorer lifestyle and behaviour that increases cognitive risk^51^. It has also been suggested that low SES may act as a proxy for reduced exposure to intellectually stimulating environments with inadequate cognitive resources to protect against neurodegeneration^9,47,52^. Biological age, as measured by telomere length is considered a composite marker of current health status encapsulating cumulative burdens of chronic comorbidities and psychosocial stress from allostatic load^13,53^. Shorter TL is associated with age-related morbidity, lower immune function, elevated oxidative stress-dependent senescence, and pro-inflammatory mediators^54^. Given the potential overlap between these two constellations of pathways, our study suggests SES and LTL were synergistic for dementia. These findings may suggest that both SES and biological aging potentially contribute to ‘downstream’ mechanisms underlying increased dementia risk. For example, inflammatory processes are increasingly implicated as systemic influences in neurodegeneration, and both biological ageing and SES may act separately to upregulate neuro-inflammatory pathways.

Previous evidence mostly showed significant associations between individual-level SES and dementia in elderly cohorts comprising late-onset cases. However, research on early-onset dementia has been relatively scarce. In our UK Biobank analytic sample, 233 of 3,313 (7%) dementia cases presented themselves as early-onset cases. Our stratified analysis by age in the study sub-groups found that the effect sizes for socioeconomic status on the risk of dementia and Alzheimer’s disease in the younger age subgroup (< 65 years) comprising early-onset cases remained significant, as were the older sub-groups (≥ 65) with late-onset cases. Joint associational analysis found that the HR for dementia in the double-exposed group (relative to the least-exposed) were 4.57 for the subgroup with early-onset cases (< 65 years) and 6.37 for subgroup with late-onset cases (≥ 65 years). In early-onset dementia (including frontotemporal dementia), symptom onset occurs at a relatively younger age when the patients are still in a crucial life stage, being inactive employment with family and social responsibilities, thereby making them vulnerable. Patients generally experience a steeper decline in cognitive and functional capacities as compared to late-onset dementia^55,56^. It is noteworthy to mention that the observed significant associations between early-onset dementia and SES are of value, and may plausibly point to the protective role of socioeconomic position (and associated lifestyle) on dementia, potentially via lowering stress and chronic inflammation. Further research needs to be conducted in this direction.

In our models, biological age was defined as a composite measure derived from residual LTL adjusting for chronological age, inflammation (CRP) and 46 curated chronic comorbidities including risk factors of dementia. Sensitivity tests in which models included residual LTL adjusted for age and serum CRP only, and additionally adjusted for the comorbidities as covariates also produced consistent results. The generalizability of our findings to other cohorts in diverse settings also needs to be explored.

With increasing proportion of elderly population and stronger impetus towards ensuring healthy longevity, evidence as generated from population stratification by SES and biological age may help identify vulnerable sub-groups for allocating crucial public health and social care resources. Risk factors such as SES may act as important considerations for community screening of neurodegenerative diseases as well as designing of tailored intervention programs to prevent against cognitive impairment. Further research is needed to improve technologies for measuring biological age (telomere length) at-scale, accuracy and cost effectiveness^57,58^.

### Strengths

Strengths of the study include scale and follow-up period. This analysis is an order of magnitude larger than previous studies linking either SES or LTL to dementia, enabling smaller effect sizes and interactions to be detected with confidence. The 12-year follow-up period, also longer than many previous studies, has enabled the analysis to focus on incident cases and implement a landmark period, to minimise the impact of reverse causation. Latent class modelling is a parsimonious approach to developing a composite SES construct. Complimentary sensitivity analyses using individual base SES variables further confirmed the primary analysis. Biological age was comprehensively measured from LTL which underwent rigorous quality checks.

### Limitations

We acknowledge several limitations. Firstly, ascertainment of dementia is a challenge for health services across the world. We defined dementia and Alzheimer’s disease through electronic health record linkage as in previous studies^9,59,60^, which would have potentially led to an underestimation through omitting undiagnosed conditions. Previous cohort studies have estimated the low sensitivities of dementia diagnosis from hospital discharge records; for example 51% in Finland^61^, 78% in England^62^ and 70% in USA^63^. A recent systematic review concluded that the positive predictive value (PPV) for all-cause dementia from routinely collected health datasets were > 75% for 16 of the 27 studies, while the sensitivities were reasonable ranging between 21 and 86%^64^. Such an under-ascertainment would be more likely among ethnic minority, older sub-groups and in participants with milder dementia and poorer health-seeking behaviour. Nonetheless, routinely collected and coded hospital records linked to large population cohorts constitute a cost-effective means of case ascertainment for dementia with minimum attrition rate in prospective analysis relative to standardized clinical assessment needing face to face examination. Within the UK Biobank, PPV for all-cause dementia from hospital admissions records remained high at 87.3%^65^. Secondly, the UK Biobank population sample is not representative. Compared to the general population, cohort participants were less likely to be sampled from socioeconomically deprived areas and had lower prevalence of obesity, smoking, drinking and self-reported health conditions due to healthy volunteer selection bias, and as such, summary statistics are not generalizable^66^. However, being a large and heterogeneous cohort, it is designed specifically for etiologic analyses and sampling bias is unlikely to have a material effect on the estimates reported^67^. Thirdly, as in any observational study, we cannot completely rule out residual confounding and reverse causation and causality cannot be assumed. However, our results remained robust subsequent to exclusion of cases over the 3-year landmark period and adjustments for chronic morbidities. Fourthly, our primary SES exposure was based on self-reported measures of household income, educational attainment and employment status. Lastly, we excluded participants due to missingness across covariates. Nonetheless, there was no systematic difference between analytic sample and excluded sample (Supplementary Table S5) and our primary results were consistent with those of sensitivity analyses using imputed data.

In conclusion, in a large prospective study we find that SES and biological aging are synergistic risk factors for dementia and Alzheimer’s disease suggesting the importance of targeted tailor-made preventive interventions in vulnerable at-risk population groups.

## Supplementary Information

Supplementary Information.

## Data Availability

Data are available from the UK Biobank (https://www.ukbiobank.ac.uk/) for researchers who meet the criteria for access to de-identified UK Biobank data.
