# Supplementary material for: The associations of socioeconomic status with incident dementia and Alzheimer’s disease are modified by leucocyte telomere length: a population-based cohort study
Source: Sci Rep. 2023 Apr 15;13:6163. doi: 10.1038/s41598-023-32974-x (PMC10105714; doi:10.1038/s41598-023-32974-x)
Supplement: Supplementary file 1 — Supplementary Information. [file 41598_2023_32974_MOESM1_ESM.docx]

**Supplementary Information**

**Table S1. Review on the association between telomere length (TL) and dementia.**

| Author | Design | Exposure | Outcome | Confounders | Main results |
| --- | --- | --- | --- | --- | --- |
| Martin-Ruiz, et al. ^1^ | 195 non-demented stroke survivors aged ≥75 years from hospital based stroke registers in Tyneside and Wearside, UK with a follow-up time of 2 years | Peripheral blood mononuclear cell (PBMC) TL (in-gel hybridization; PCR) | Dementia - according to *Diagnostic*  *and Statistical Manual of Mental Disorders*, Fourth Edition (DSM-IV) criteria | age, cardiac failure, myocardial infarction, angina, hypertension,  atrial fibrillation, ApoE4 status | Longer telomeres at baseline were associated with reduced risk for dementia (OR= 0.19; 95% CI, 0.07– 0.54 for every 1000 base pair increase in TL) |
| Honig, et al. ^2^ | 257 individuals from the Washington Heights – Inwood Community Aging Project in New York city aged 66-103 years followed up over 18 months | Leucocyte TL (PCR) | Dementia was determined  based on Diagnostic and Statistical  Manual (of Mental Disorders), Fourth Edition, Text Revised  (DSM-IV-TR) criteria and National Institute of Neurological Disorders and Stroke-Alzheimer’s Disease and Related Disorders Association criteria for AD | age, sex, ethnic group, and level of education | Mean TL was shorter among participants with Alzheimer’s disease compared with those without dementia. Relative to participants with AD and having longest TL, higher odds of mortality was reported in those with intermediate TL (OR=4.8, 1.7-13.8) and shortest TL (OR=7.3, 2.4-22.0). |
| Thomas, et al. ^3^ | 54 clinically diagnosed Alzheimer’s patients age 58–93 years and 56 controls aged between 18 and 75 from Adelaide, Australia | TL in buccal cell, white blood cells and brain tissue (PCR) | Alzheimer’s disease – based on criteria outlined by the National Institute of Neurological & Communicative Disorders and Stroke-Alzheimer’s Disease and related Disorders Association (NINCDS-AD&DA) |  | Significantly shorter TL in buccal cell and white blood cells was reported among Alzheimer’s disease patients relative to healthy age-matched controls. There was higher odds of being diagnosed with Alzheimer’s disease among participants with white blood cell TL<115 kb per diploid genome (OR=10.8, 1.19-97.85) |
| Zekry, et al. ^4^ | 439 subjects with a mean age of 85.1 years in Switzerland | Measurement of TL in peripheral blood lymphocytes (ﬂow cytometry) | Dementia severity - assessed with the Clinical Dementia Rating Scale | ApoE polymorphism and age | No signiﬁcant differences in TL were found between patients with different etiologies or severities of dementia |
| Movérare-Skrtic, et al. ^5^ | Patients with AD or mild cognitive impairment (MCI) diagnosed with AD upon follow-up (n=32),  patients with stable MCI (n=13), patients with other dementias diagnosed at primary evaluation or upon follow-up (n= 15), and healthy controls (n=20) with a median follow-up period of 3 years | Leukocyte TL (PCR) | AD - fulfilled the criteria of dementia and the criteria of AD defined by NINCDS-ADRDA |  | Patients with MCI that later converted to AD had similar LTL as patients with clinical diagnosis of AD at primary evaluation and healthy controls whereas the LTL was longer compared to the stable MCI group |
| Honig, et al. ^6^ | 1,973 subjects aged ≥65 years from the Washington Heights – Inwood Community Aging Project in New York city with a mean follow-up time of 7.8 years | Leucocyte TL (PCR) | Dementia - diagnosed by using *Diagnostic and Statistical Manual of Mental Disorders* (Fourth Edition, Text Revision) dementia criteria and the National Institute of Neurological and Communicative Disroders and Stroke-Alzheimer’s Disease and Related Disorder Association criteria for AD | age at blood draw, sex, ethnicity, education, and APOE ε4 carrier status | Individuals who developed dementia during the follow-up had significantly shorter TL than those free from dementia. Each kilobase pair attrition in TL was associated with 21% higher risk of dementia (HR=1.21, 1.00-1.46) |
| Degerman, et al. ^7^ | 168 patients with idiopathic parkinsonism (136 Parkinson’s disease; 17 Progressive Supranuclear Palsy; 15 Multiple System Atrophy) and 30 controls in northern Sweden with a follow-up time of 3 years | TL (PCR) | Dementia - based on all available information including medical files, neuropsychological testing, temporal cognitive decline detected either by the repeated neuropsychological evaluations, interview by the study nurse to detect functional decline or information from family members | NA | Among patients with Parkinson’s disease (PD) and progressive supranuclear palsy (PSP), relative TL was longer in participants who developed dementia within 3 years (p=0.007 for PD and p=0.037 for PSP). |
| Tedone, et al. ^8^ | Retrospective study: 31 late-onset AD (20 had slow-progressing decline in Mini Mental State Examination (MMSE) and 11 had fast progressing decline in MMSE) and 20 age-matched healthy elderly in Milan or Paris with a follow-up period of 2 years | TL (ﬂow cytometry) | AD patients were categorized using criteria as in NINCDS-ADRDA | NA | AD patients who had slow-progressing decline in Mini Mental State Examination (MMSE) showed shorter TL as compared with healthy elderly controls (p=0.034). |
| Koh, et al. ^9^ | 28 patients with mild cognitive impairment and 104 cognitively unimpaired individuals without amyloid pathology as controls in Korea with a follow-up time of 2 years | Leukocyte TL | Conversion from MCI to dementia over time - dementia diagnosis based on DSM-IV-TR criteria | age | Among subjects with mild cognitive impairment with presence of amyloid pathology (A+), participants with lower TL (kb≤6.61) was associated with higher risk of dementia (HR 13.16, 95% CI, 1.11-156.61), as compared to those with higher TL (kb=7.85<). |
| Fani, et al. ^10^ | 1,961 subjects from the Rotterdam Study in Netherlands with a mean age of 71.4 and with a median follow-up time of 8.3 years | Leucocyte TL (PCR) | Dementia and Alzheimer’s disease - according to standard criteria for dementia (DSM-III-R) and Alzheimer’s disease (NINCDS–ADRDA) | age, sex, visit, education, APOE ε4  carrier status, smoking, body mass index, hypertension, hypercholesterolemia, and white blood cell count | U-shaped association with higher risks of Alzheimer’s disease in the lowest tertile of TL (HR=1.59; 1.13–2.23) as well as the highest tertile (HR=1.47; 1.03–2.10) relative to the middle tertile of TL taken as reference. |
| Ashrafi, et al. ^11^ | 1,613 cognitively unimpaired individuals as well as 597 individuals with dementia or incipient dementia from Life Long Family Study in the USA and Denmark, aged >60 years and demonstrating exceptional survival using the family longevity selection score | Leukocyte TL (PCR) | Dementia algorithm variable (National Alzheimer’s Coordinating Center (NACC)) was used to separate cognitively impaired individuals from unimpaired individuals | sex, age, education, country, generation, and lymphocyte percentage | Null association between LTL and working or episodic memory, semantic processing, and information processing speed for the cognitively unimpaired sub-group as well as individuals with dementia or incipient dementia in cross sectional analysis |
| Rodríguez-Fernández, et al. ^12^ | Two-sample Mendelian randomization analysis | Summary statistics from genome-wide meta-analysis of leukocyte TL across large-scale European-descent studies including  up to n=78,592 individuals | Summary level data from recent largescale GWAS on neurodegenerative diseases including Alzheimer’s disease (n=455,258) |  | Longer telomeres were inferred to have a protective effect on risk of Alzheimer’s disease (OR = 0.964, q-value 0.039) |

**Abbreviations**: PCR, polymerase chain reaction; NINCDS-ADRDA, National Institute of Neurological and Communicative Disorders and Stroke-Alzheimer’s Disease and related Disorders Association.

**References**

1 Martin-Ruiz, C. *et al.* Telomere length predicts poststroke mortality, dementia, and cognitive decline. *Annals of Neurology* **60**, 174-180, doi:<https://doi.org/10.1002/ana.20869> (2006).

2 Honig, L. S., Schupf, N., Lee, J. H., Tang, M. X. & Mayeux, R. Shorter telomeres are associated with mortality in those with APOE ϵ4 and dementia. *Annals of Neurology* **60**, 181-187, doi:<https://doi.org/10.1002/ana.20894> (2006).

3 Thomas, P., O’ Callaghan, N. J. & Fenech, M. Telomere length in white blood cells, buccal cells and brain tissue and its variation with ageing and Alzheimer's disease. *Mechanisms of Ageing and Development* **129**, 183-190, doi:<https://doi.org/10.1016/j.mad.2007.12.004> (2008).

4 Zekry, D. *et al.* Telomere length and ApoE polymorphism in mild cognitive impairment, degenerative and vascular dementia. *Journal of the Neurological Sciences* **299**, 108-111, doi:10.1016/j.jns.2010.07.019 (2010).

5 Movérare-Skrtic, S. *et al.* Leukocyte Telomere Length (LTL) is reduced in stable mild cognitive impairment but low LTL is not associated with conversion to Alzheimer's Disease: A pilot study. *Experimental Gerontology* **47**, 179-182, doi:<https://doi.org/10.1016/j.exger.2011.12.005> (2012).

6 Honig, L. S., Kang, M. S., Schupf, N., Lee, J. H. & Mayeux, R. Association of Shorter Leukocyte Telomere Repeat Length With Dementia and Mortality. *Archives of Neurology* **69**, 1332-1339, doi:10.1001/archneurol.2012.1541 (2012).

7 Degerman, S. *et al.* Long Leukocyte Telomere Length at Diagnosis Is a Risk Factor for Dementia Progression in Idiopathic Parkinsonism. *PLOS ONE* **9**, e113387, doi:10.1371/journal.pone.0113387 (2014).

8 Tedone, E. *et al.* Leukocyte Telomere Length in Alzheimer’s Disease Patients with a Different Rate of Progression. *Journal of Alzheimer's Disease* **46**, 761-769, doi:10.3233/JAD-142808 (2015).

9 Koh, S.-H. *et al.* Telomere shortening reflecting physical aging is associated with cognitive decline and dementia conversion in mild cognitive impairment due to Alzheimer&#x2019;s disease. *Aging* **12**, 4407-4423, doi:10.18632/aging.102893 (2020).

10 Fani, L. *et al.* Telomere Length and the Risk of Alzheimer’s Disease: The Rotterdam Study. *Journal of Alzheimer's Disease* **73**, 707-714, doi:10.3233/JAD-190759 (2020).

11 Ashrafi, A. *et al.* Leukocyte Telomere Length Is Unrelated to Cognitive Performance Among Non-Demented and Demented Persons: An Examination of Long Life Family Study Participants. *Journal of the International Neuropsychological Society* **26**, 906-917, doi:10.1017/S1355617720000363 (2020).

12 Rodríguez-Fernández, B. *et al.* Genetically predicted telomere length and its relationship with neurodegenerative diseases and life expectancy. *Computational and Structural Biotechnology Journal* **20**, 4251-4256, doi:<https://doi.org/10.1016/j.csbj.2022.08.006> (2022).

**Methods S1**

**Procedures of latent class analysis**

Latent Class Analysis (LCA)^13^, a methodology widely used in medicine, psychology and social science research, was conducted to develop a composite metrics of individual-level socioeconomic status (SES) based on multiple individual socioeconomic factors of the UK Biobank participants. Three individual socioeconomic factors were taken as inputs in the latent class model, namely highest education attainment (coded as seven-category variable), employment status (as a three-category variable) and average total household income before tax (as a five-category variable) were taken as inputs in the latent class model^14^.

Maximum likelihood method, which permits the use of all data available including those with missingness, was employed in the latent class analysis. Iterations were undertaken to check the convergence and performance of the latent class models for two, three and four or more classes. The final model was selected based on fit statistics parameters of Bayesian information criteria (BIC) and Akaike information criterion (AIC). Based on the AIC and BIC values, we selected the latent class model with three classes to define individual-level SES for this study. The mean posterior probability values of the latent class model with three classes are 0.73, 0.81 and 0.84 for classes 1, 2 and 3 respectively.

In the latent class model with three classes (Supplementary Table S1 below), participants in latent class 1 were characterized by fair education attainment (such as other professional, NVQ/HND/HNC, CSEs), higher proportion of being employed and having a household income in the range between £18,000 and £51,999. We defined participants in latent class 1 as having medium individual-level SES. In latent class 2, participants were mostly characterized by lower education attainment (such as CSEs, none of the above), lower proportion of being employed and lower household income (having a household income of less than £18,000). We therefore defined participants in latent class 2 as having low individual-level SES. In latent class 3, the proportion of participants obtaining other professional education, being employed and having household income in the range of £52,000 – £100,000 and above was relatively higher in latent class 3. Hence, we defined participants in latent class 3 as having high individual-level SES.

**Table S2. Latent class marginal means for latent class models with two and three classes by education qualification, employment status and household income.**

|  | **Latent class model** | | | | |
| --- | --- | --- | --- | --- | --- |
|  | **Two classes** | | **Three classes** | | |
|  | 1 | 2 | 1 | 2 | 3 |
|  | Mean^a^ | Mean^a^ | Mean^a^ | Mean^a^ | Mean^a^ |
| **Educational qualification** | | | | | |
| Other professional | 0.47 | 0.15 | 0.27 | 0.13 | 0.58 |
| NVQ/HND/HNC | 0.14 | 0.08 | 0.13 | 0.07 | 0.13 |
| CSEs | 0.20 | 0.23 | 0.28 | 0.20 | 0.15 |
| O levels/GCSEs | 0.06 | 0.05 | 0.08 | 0.05 | 0.03 |
| A levels/AS levels | 0.06 | 0.08 | 0.08 | 0.07 | 0.04 |
| College or University degree | 0.05 | 0.06 | 0.06 | 0.06 | 0.04 |
| None of the above | 0.04 | 0.35 | 0.11 | 0.41 | 0.02 |
|  |  |  |  |  |  |
| **Employment status** |  |  |  |  |  |
| Employed/self-employed | 0.74 | 0.28 | 0.61 | 0.21 | 0.77 |
| Retired | 0.22 | 0.57 | 0.36 | 0.60 | 0.17 |
| Others | 0.04 | 0.15 | 0.03 | 0.20 | 0.06 |
|  |  |  |  |  |  |
| **Household income** |  |  |  |  |  |
| <£18,000 | 4.52x10^-6^ | 0.56 | 0.04 | 0.73 | 0.02 |
| £18,000 – £30,999 | 0.18 | 0.36 | 0.50 | 0.24 | 5.48x10^-6^ |
| £31,000 – £51,999 | 0.38 | 0.08 | 0.42 | 0.02 | 0.28 |
| £52,000 - £100,000 | 0.34 | 8.03x10^-7^ | 0.04 | 0.01 | 0.55 |
| ≥£100,000 | 0.09 | 1.41x10^-8^ | 1.53x10^-8^ | 0.00 | 0.15 |

^a^Latent class marginal means

**Methods S2**

**Additional information about covariates**

Socio-demographic covariates were obtained via self-reported structured questionnaires during the participants’ visits to the assessment centre.

- Age was calculated from the date of birth to the date of baseline assessment.
- Ethnicity was assessed by the question on ethnicity with options including: 'White', 'British', 'White and Black Caribbean', 'Indian', 'Caribbean', 'Mixed', 'Irish', 'White and Black African', 'Pakistani', 'African', 'Asian or Asian British', 'Any other white background', 'White and Asian', 'Bangladeshi', 'Any other Black background', 'Black or Black British', 'Any other mixed background', 'Any other Asian background', 'Chinese', 'Other ethnic group', 'Do not know', 'Prefer not to answer'.
- Healthy lifestyle score: was calculated based on three components including smoking status, alcohol intake frequency and diet.

1. Smoking status was derived from a questionnaire where the participants were asked to summarize current/past smoking status with options including ‘Current’, ‘Previous’, ‘Never’ and ‘Prefer not to answer’.
2. Alcohol intake frequency was derived from a questionnaire on intake frequency where the participants were asked to choose between ‘Daily or almost daily’, ‘Three or four times a week’, ‘Once or twice a month’, ‘One to three times a month’, ‘Special occasions only’, ‘Never’ or ‘Prefer not to answer’.
3. Diet was evaluated based on self-reported consumption of seven items, namely, fruits, vegetables, fish, processed meats, unprocessed red meats, whole grains and refined grains. Participants were categorized as healthy if they fulfilled at least 4 of the following 7 dietary criteria.

- Fruits consumption was evaluated through questionnaires on fresh fruit intake (‘About how many pieces of FRESH fruit would you eat per DAY?’) and dried fruits intake (‘About how many pieces of DRIED fruit would you eat per DAY?’). Participants achieved intake goal in this dietary component if they consumed ≥3 servings of fruits daily.
- Vegetable consumption was measured from questionnaires on cooked vegetable intake (‘On average how many heaped tablespoons of COOKED vegetables would you eat per DAY?’) and salad/raw vegetable intake (‘On average how many heaped tablespoons of SALAD or RAW vegetables would you eat per DAY?’). Participants achieved intake goal in this dietary component if they consumed ≥3 servings of vegetables daily.
- Fish consumption was measured by questionnaires on oily fish intake (‘How often do you eat oily fish?’ with options including ‘Never’, ‘Less than once a week’, ‘Once a week’, ‘2-4 times a week’, ‘5-6 times a week’, ‘Once or more daily’, ‘Do not know’ and ‘Prefer not to answer’) and non-oily fish intake (‘How often do you eat other types of fish?’ with options including ‘Never’, ‘Less than once a week’, ‘Once a week’, ‘2-4 times a week’, ‘5-6 times a week’, ‘Once or more daily’, ‘Do not know’ and ‘Prefer not to answer’). Participants achieved intake goal in this dietary component if they consumed ≥2 servings of fish weekly.
- Processed meats consumption was evaluated from questionnaires on processed meats intake (‘How often do you eat processed meats (such as bacon, ham, sausages, meat pies, kebabs, burgers, chicken nuggets)?’ with options including ‘Never’, ‘Less than once a week’, ‘Once a week’, ‘2-4 times a week’, ‘5-6 times a week’, ‘Once or more daily’, ‘Do not know’ and ‘Prefer not to answer’). Participants achieved intake goal in this dietary component if they consumed ≤1 serving of processed meats weekly.
- Unprocessed red meats consumption was evaluated based on questionnaires about beef (‘How often do you eat beef?’), lamb/mutton (‘How often do you eat lamb/mutton?’) and pork intake (‘How often do you eat pork?’). Options for each of the question included ‘Never’, ‘Less than once a week’, ‘Once a week’, ‘2-4 times a week’, ‘5-6 times a week’, ‘Once or more daily’, ‘Do not know’ and ‘Prefer not to answer’. Participants achieved intake goal in this dietary component if they consumed ≤1.5 serving of unprocessed red meats weekly.
- Whole grains consumption was evaluated from questionnaires on bread intake (‘How many slices of bread do you eat each WEEK?’), bread type (‘What type of bread do you mainly eat?’ with options including ‘White’, ‘Brown’, ‘Wholemeal or whole grain’, ‘Other type of bread’, ‘Do not know’, ‘Prefer not to answer’), cereal intake (‘How many bowls of cereal do you eat a WEEK?’), and cereal type (‘What type of cereal do you mainly eat?’ with options including ‘Bran cereal’, ‘Biscuit cereal’, ‘Oat cereal’, ‘Muesli’, ‘Other’, ‘Do not know’ and ‘Prefer not to answer’). Participants achieved intake goal in this dietary component if they consumed ≥3 servings of whole grains daily.
- Refined grains consumption was evaluated from questionnaires on bread intake (‘How many slices of bread do you eat each WEEK?’), bread type (‘What type of bread do you mainly eat?’ with options including ‘White’, ‘Brown’, ‘Wholemeal or whole grain’, ‘Other type of bread’, ‘Do not know’, ‘Prefer not to answer’), cereal intake (‘How many bowls of cereal do you eat a WEEK?’), and cereal type (‘What type of cereal do you mainly eat?’ with options including ‘Bran cereal’, ‘Biscuit cereal’, ‘Oat cereal’, ‘Muesli’, ‘Other’, ‘Do not know’ and ‘Prefer not to answer’). Participants achieved intake goal in this dietary component if they consumed ≤1.5 servings of refined grains daily.

A score of 1 was assigned to participants who achieved the healthy standard in each lifestyle component (non-current smoker; alcohol not consumed daily/almost daily; consumed ≥4 of 7 types of food following dietary recommendations as previously). The healthy score ranged between 0 and 3 was subsequently categorized as favorable (a score of 3), intermediate (a score of 2) and unfavorable (a score of 0 or 1) following the data distribution.

- Number of leisure and social activities in a week: This was derived from the question, 'Which of the following do you attend once a week or more often? (You can select more than one)', with the option to select from: 'Sports club or gym', 'Pub or social club', 'Religious group', 'Adult education class', 'Other group activity', 'Do not know', 'Prefer not to answer'.

**Additional information on clinical variables**

- Abdominal obesity: This was defined in terms of waist-hip ratio (WHR) ≥0.85 cm for women and ≥0.90 cm. The waist-hip ratio was measured by using a Seca 200 measuring tape.
- Frailty: Frailty was assessed at baseline from five phenotypes; weight loss, exhaustion, low grip strength, low physical activity and slow gait speed and coded as non-frail, frail/pre-frail^15,16^.

(1) Weight loss was derived from the questionnaire on weight change, ‘Compared with one year ago, has your weight changed?', with the option to select from: 'No - weigh about the same', 'Yes - gained weight', 'Yes - lost weight', 'Do not know', 'Prefer not to answer'.

(2) Low gait speed was derived from the questionnaire, 'How would you describe your usual walking pace?', with the option of selecting one from: 'Slow pace', 'Steady average pace', 'Brisk pace', 'None of the above', and 'Prefer not to answer' which was recoded as a binary variable.

(3) Grip strength was measured with the use of a Jamar J00105 hydraulic hand dynamometer. Participants were asked to sit upright in a chair and squeeze the handle of the dynamometer as strongly as they can with the use of their left hand for approximately 3 seconds. The grip strength measurement was then repeated by using their right hand. The higher value of the two grip strength measurements was used in the analysis. Low grip strength was defined based on sex and body-mass index adjusted thresholds (Men: ≤29 for BMI≤24, ≤30 for BMI≥24.1 and ≤28, ≤32 for BMI>28; Women: ≤17 for BMI≤23, ≤17.3 for BMI≥23.1 and ≤26, ≤18 for BMI≥26.1 and ≤29, ≤21 for BMI>29).

(4) Exhaustion was evaluated based on the question ‘Over the past two weeks, how often have you felt tired or had little energy?’ with the option to select from ‘not at all’, ‘Several days’, ‘More than half the days’, ‘Nearly every day’, ‘Do not know’, ‘Prefer not to answer’. Participants who answered ‘More than half the days’ or ‘Nearly every day’ met the criteria of exhaustion.

(5) Low physical activity was evaluated from the question, ‘In the last 4 weeks did you spend any time doing the following? (You can select more than one answer)’. The responses were classified as none (no physical activity), low (light DIY activities such as pruning, watering the lawn), medium (including heavy DIY activities (such as weeding, lawn mowing, carpentry, digging), walking for pleasure or other exercises) and high (strenuous sports). Participants who conducted non or light physical activity over the past 4 weeks met the criteria of low physical activity. Participants who did not meet any of the five frailty criteria were defined as not frail, while meeting any one or two were classified as pre-frail and three or more as frail.

- Serum CRP value expressed as mg/L was measured by immunoturbidimetric high-sensitivity analysis with the use of a Beckman Coulter AU5800 ^17^
- Hearing difficulty: This was derived from the question, “Do you have any difficulty with your hearing?”, with the option to choose from: ‘Yes’, ‘No’, ‘I am completely deaf’, ‘Do not know’, ‘Prefer not to answer’.

**Other variables for sensitivity analyses**

- Number of household members: This was derived from the question, “Including yourself, how many people are living together in your household? (Include those who usually live in the house such as students living away from home during term, partners in the armed forces or professions such as pilots)”. Special values include “Do not know” and “Prefer not to answer”.
- Job: This was classified into categories including “Managers and Senior Officials”, “Professional Occupations”, “Associate Professional and Technical Occupations”, “Administrative and Secretarial Occupations”, “Skilled Trades Occupations”, “Personal Service Occupations”, “Personal Service Occupations”, “Process, Plant and Machine Operatives”, or “Elementary Occupations”.

**Additional information on neighbourhood environment**

- Urbanicity was obtained from the UK Biobank Urban Morphometric platform, a built environment exposure database we had developed for measuring urban exposures^18^. Participants’ residential addresses were first geocoded and neighbourhoods comprising street catchments of 1.0 kilometers were delineated. As in our previous studies^19^, a composite index of urbanicity was defined as:

${Urbanicity}_{i}^{1 Km}={zscore}_{HousingD}+ {zscore}_{RetailD}+ {zscore}_{PublicTransD}+ {zscore}_{Centrality}$

…………… (6)

where ${Urbanicity}_{i}^{1 Km}$ is the urbanicity index for a cohort participant ‘i’ within an 1 Km street catchment neighbourhood comprising z-standardized components for housing density (*HousingD*), retail density (*RetailD*), and public transport (*PublicTransD*) and urban centrality (*Centrality*). These metrics of density and urbanicity were measured within a neighbourhood of 1 Km residential street catchment and were available for majority of the UK Biobank participants whose residential addresses could be geocoded, n=480,503 (95.6%).

- Neighbourhood deprivation was measured by the Townsend deprivation index, a composite measure from four components; unemployment rate, overcrowding, non-car ownership and non-home ownership measured for each output areas (census aggregate) and derived from the 2011 national census data. The output area is the lowest geographical level of aggregated census data. Each output area is equivalent to a minimum of 40 households for England and Wales, and 20 in Scotland. Participants were assigned a score linked via their postcode of residence.

**References**

1 Martin-Ruiz, C. *et al.* Telomere length predicts poststroke mortality, dementia, and cognitive decline. *Annals of Neurology* **60**, 174-180, doi:<https://doi.org/10.1002/ana.20869> (2006).

2 Honig, L. S., Schupf, N., Lee, J. H., Tang, M. X. & Mayeux, R. Shorter telomeres are associated with mortality in those with APOE ϵ4 and dementia. *Annals of Neurology* **60**, 181-187, doi:<https://doi.org/10.1002/ana.20894> (2006).

3 Thomas, P., O’ Callaghan, N. J. & Fenech, M. Telomere length in white blood cells, buccal cells and brain tissue and its variation with ageing and Alzheimer's disease. *Mechanisms of Ageing and Development* **129**, 183-190, doi:<https://doi.org/10.1016/j.mad.2007.12.004> (2008).

4 Zekry, D. *et al.* Telomere length and ApoE polymorphism in mild cognitive impairment, degenerative and vascular dementia. *Journal of the Neurological Sciences* **299**, 108-111, doi:10.1016/j.jns.2010.07.019 (2010).

5 Movérare-Skrtic, S. *et al.* Leukocyte Telomere Length (LTL) is reduced in stable mild cognitive impairment but low LTL is not associated with conversion to Alzheimer's Disease: A pilot study. *Experimental Gerontology* **47**, 179-182, doi:<https://doi.org/10.1016/j.exger.2011.12.005> (2012).

6 Honig, L. S., Kang, M. S., Schupf, N., Lee, J. H. & Mayeux, R. Association of Shorter Leukocyte Telomere Repeat Length With Dementia and Mortality. *Archives of Neurology* **69**, 1332-1339, doi:10.1001/archneurol.2012.1541 (2012).

7 Degerman, S. *et al.* Long Leukocyte Telomere Length at Diagnosis Is a Risk Factor for Dementia Progression in Idiopathic Parkinsonism. *PLOS ONE* **9**, e113387, doi:10.1371/journal.pone.0113387 (2014).

8 Tedone, E. *et al.* Leukocyte Telomere Length in Alzheimer’s Disease Patients with a Different Rate of Progression. *Journal of Alzheimer's Disease* **46**, 761-769, doi:10.3233/JAD-142808 (2015).

9 Koh, S.-H. *et al.* Telomere shortening reflecting physical aging is associated with cognitive decline and dementia conversion in mild cognitive impairment due to Alzheimer&#x2019;s disease. *Aging* **12**, 4407-4423, doi:10.18632/aging.102893 (2020).

10 Fani, L. *et al.* Telomere Length and the Risk of Alzheimer’s Disease: The Rotterdam Study. *Journal of Alzheimer's Disease* **73**, 707-714, doi:10.3233/JAD-190759 (2020).

11 Ashrafi, A. *et al.* Leukocyte Telomere Length Is Unrelated to Cognitive Performance Among Non-Demented and Demented Persons: An Examination of Long Life Family Study Participants. *Journal of the International Neuropsychological Society* **26**, 906-917, doi:10.1017/S1355617720000363 (2020).

12 Rodríguez-Fernández, B. *et al.* Genetically predicted telomere length and its relationship with neurodegenerative diseases and life expectancy. *Computational and Structural Biotechnology Journal* **20**, 4251-4256, doi:<https://doi.org/10.1016/j.csbj.2022.08.006> (2022).

13 Lanza, S. T. & Rhoades, B. L. Latent Class Analysis: An Alternative Perspective on Subgroup Analysis in Prevention and Treatment. *Prevention Science* **14**, 157-168, doi:10.1007/s11121-011-0201-1 (2013).

14 Zhang, Y.-B. *et al.* Associations of healthy lifestyle and socioeconomic status with mortality and incident cardiovascular disease: two prospective cohort studies. *BMJ* **373**, n604, doi:10.1136/bmj.n604 (2021).

15 Fried, L. P. *et al.* Frailty in Older Adults: Evidence for a Phenotype. *The Journals of Gerontology: Series A* **56**, M146-M157, doi:10.1093/gerona/56.3.M146 (2001).

16 Hanlon, P. *et al.* Frailty and pre-frailty in middle-aged and older adults and its association with multimorbidity and mortality: a prospective analysis of 493 737 UK Biobank participants. *The Lancet Public Health* **3**, e323-e332, doi:<https://doi.org/10.1016/S2468-2667(18)30091-4> (2018).

17 Fry, D., Almond, R., Moffat, S., Gordon, M. & Singh, P. UK Biobank Biomarker Project Companion Document to Accompany Serum Biomarker Data. Version 1., (2020).

18 Sarkar, C., Webster, C. & Gallacher, J. UK Biobank Urban Morphometric Platform (UKBUMP) – a nationwide resource for evidence-based healthy city planning and public health interventions. *Annals of GIS* **21**, 135-148, doi:10.1080/19475683.2015.1027791 (2015).

19 Sarkar, C. *et al.* Environmental correlates of chronic obstructive pulmonary disease in 96 779 participants from the UK Biobank: a cross-sectional, observational study. *The Lancet Planetary Health* **3**, e478-e490, doi:<https://doi.org/10.1016/S2542-5196(19)30214-1> (2019).

**Table S3. International Classification of Diseases (ICD) 10 codes used in the UK Biobank for identification of incident clinical cases of dementia and Alzheimer’s disease from linked hospital records.**

| Category | Description | ICD-10 codes |
| --- | --- | --- |
| All-cause dementia | Dementia in Alzheimer disease | F00 |
|  | Vascular dementia | F01 |
|  | Dementia in other diseases classified elsewhere | F02 |
|  | Unspecified dementia | F03 |
|  | Delirium superimposed on dementia | F05.1 |
|  | Mental and behavioural disorders due to use of alcohol - Amnesic syndrome | F10.6 |
|  | Alzheimer’s disease | G30 |
|  | Other degenerative diseases of nervous system, not elsewhere classified |  |
|  | Circumscribed brain atrophy | G31 |
|  | Senile degeneration of brain | G31.1 |
|  | Other specified degenerative diseases of  nervous system | G31.8 |
|  | Lewy body(ies) dementia disease | G31.83 |
|  | Creutzfeldt-Jakob disease | A81.0 |
|  |  |  |
| Alzheimer’s disease | Dementia in Alzheimer disease | F00 |
|  | Alzheimer’s disease | G30 |

**Table S4. Definitions of prevalent cases of 46 curated diseases at baseline using UK Biobank self-reported case codes, ICD9 and ICD10 codes from linked hospital records to correctly identify cases.**

| Category | Sub-category | UK Biobank ICD-9 codes | UK Biobank ICD-10 codes | UK Biobank self-reported case codes ^a^ |
| --- | --- | --- | --- | --- |
| Cancer | Bladder | 188 | C67 | Bladder cancer (20001=1035) |
|  | Brain | 191 | C70-C72, C75.1-C75.4 | Brain cancer/primary malignant brain tumour (20001=1032) |
|  | Breast | 174 | C50, D05 | Breast cancer (20001=1002) |
|  | Cervical | 180 | C53 | Cervical cancer (20001=1041), cin/pre-cancer cells cervix (20001=1072) |
|  | Colorectal | 153, 154 | C18-C21 | Large bowel cancer/colorectal cancer (20001=1020), anal cancer (20001=1021), colon cancer/sigmoid cancer (20001=1022), rectal cancer (20001=1023) |
|  | Head and neck | 140-149, 160-161 | C00-C14, C30-C32 | Cancer of lip/mouth/pharynx/oral cavity (20001:1004), larynx/throat cancer (20001=1006), nasal cavity cancer (20001=1007), sinus cancer (20001=1009), lip cancer (20001=1010), tongue cancer (20001=1011), gum cancer (20001=1012), parotid gland cancer (20001=1015), other salivary gland cancer (20001=1016), mouth cancer (20001=1077), tonsil cancer (20001=1078), oropharynx/oropharyngeal cancer (20001=1079) |
|  | Kidney | 189 | C64-C66 | Kidney/renal cell cancer (20001=1034) |
|  | Leukemia | 204-208 | C91-C95 | Leukemia (20001=1048), chronic lymphocytic(20001=1055), chronic myeloid (20001=1056), acute myeloid leukemia (20001=1074) |
|  | Liver | 155 | C22 | Liver/hepatocellular cancer (20001=1024) |
|  | Lung | 162 | C34 | Lung cancer (20001=1001), small cell lung cancer (20001=1027), non-small cell lung cancer (20001=1028) |
|  | Lymphoma | 200-203 | C81-C86, C88, C90 | Lymphoma (20001=1047), multiple myeloma (20001=1050), Hodgkin’s lymphoma/Hodgkin’s disease (20001=1052), non-Hodgkin’s lymphoma (20001=1053) |
|  | Melanoma | 172 | C43 | Malignant melanoma (20001=1059) |
|  | Oesophageal | 150 | C15 | Oesophageal cancer (20001=1017) |
|  | Ovarian | 183 | C56, C570 | Ovarian cancer (20001=1039), fallopian tube cancer (20001=1087) |
|  | Pancreas | 157 | C25 | Pancreas cancer (20001=1026) |
|  | Prostate | 185 | C61 | Prostate cancer (20001=1044) |
|  | Sarcoma | 171 | C49 | Sarcoma/fibrosarcoma (20001=1068) |
|  | Small intestine | 152 | C17 | Small intestine/small bowel cancer (20001=1019) |
|  | Stomach | 151 | C16 | Stomach cancer (20001=1018) |
|  | Testicular | 186 | C62 | Testicular cancer (20001=1045) |
|  | Thyroid | 193 | C73 | Thyroid cancer (20001=1065) |
|  | Uteri | 179, 182 | C54-C55 | Uterine/endometrial cancer (20001=1040) |
|  |  |  |  |  |
| Cardiovascular | Aortic valve stenosis | 424 | I350 | Aortic stenosis (20002=1490) |
|  | Atrial fibrillation | 427.3 | I48 | atrial fibrillation (20002=1471), (20002=1483) |
|  | Coronary artery disease | 410-414 | I20-I25 | Heart attack/myocardial infarction (20002=1075), heart attack diagnosed by a doctor (6150=1/2) |
|  | Heart failure | 428 | I11, I13.0, I13.2, I50 | Heart failure/pulmonary odema (20002=1076) |
|  | Peripheral vascular disease | 443-444 | I73.9, I74 | Peripheral vascular disease (20002=1067), leg claudication/ intermittent claudication (20002=1087) |
|  | Raynauds | 443 | I73 | Raynaud's phenomenon/disease (20002=1561) |
|  | Stroke | 430-434 | I60-I64 | Ischaemic stroke (20002=1583), stroke (20002=1081), subarachnoid haemorrhage (20002=1086), vascular/heart problems diagnosed by doctor - stroke (6150=3) |
|  | Varicose veins | 454-455 | I83-I84 | Varicose veins (20002=1494), varicose ulcer (20002=1593) |
|  | Venous thromboembolism | 415.1, 451-453 | I26, I63.6, I67.6, I74, I80-I82 | Venous thromboembolic disease (20002=1068), pulmonary embolism (20002=1093), deep venous thrombosis (DVT) (20002=1094) |
| Hypertension |  | 401-405 | I10-I13, I15 | Hypertension (20002=1065), essential hypertension (20002=1072), blood pressure medications (6177=2 or 6153=2), vascular/heart problems diagnosed by doctor - high blood pressure (6150=4) |
| Diabetes | Type 1 diabetes | 250.01, 250.11, 250.21, 250.91 | E10 | Type 1 diabetes (20002=1222) |
|  | Type 2 diabetes | 250, 250.0, 250.00, 250.1, 250.10, 250.2, 250.20, 250.3-250.7, 250.9, 250.90 | E11, E13, E14 | Type 2 diabetes (20002=1223), diabetes (20002=1220) |
| Psychiatric disorder | Depression | 296.2-296.3 | F32-F33 | Depression (20002=1286) |
|  | Bipolar disorder | 296.0-296.1, 296.4-296.6, 296.8 | F30-F31 | Mania/bipolar disorder/manic depression (20002=1291) |
| Respiratory disease | Asthma | 493 | J45-J46 | Asthma (20002=1111) |
|  | COPD | 490-492, 495-496 | J40-J44 | Chronic obstructive airways disease (20002=1112), emphysema/chronic bronchitis (20002=1113) |
|  | Hay fever | 477 | J30-J31 | Hay fever/allergic rhinitis (20002=1387) |
|  | Nasal polyps | 471 | J33 | Nasal polyps (20002=1417) |
|  | Otitis media | 381-382 | H65-H66 | Not self-reported |
|  | Pleurisy | 511.0-511.1 | R09.1 | Pleurisy (20002=1125) |
|  | Pneumonia | 466, 480-487 | J10-J18, J20-J22 | Pneumonia (20002=1398) |
|  | Pneumothorax | 512, 512.9 | J93.0-J93.1, J93.8-J93.9 | Spontaneous pneumothorax/recurrent pneumothorax (20002=1126) |
|  | Sinusitis | 461, 473 | J01, J32 | Chronic sinusitis (20002=1416) |
|  | Sleep apnoea | 780.5 | G47.3 | Sleep apnoea (20002=1123) |
|  | Tonsillitis | 463, 474-475 | J03, J35 | Tonsillitis (20002=1598) |

^a^The codes represent specific disease under data fields 20001 for cancer and 20002 for non-cancer illness. If the participant was uncertain of the type of illness they had had, then they described it to a trained nurse who placed it within the coding tree.

**Table S5. Baseline characteristics of included and excluded UK Biobank participants. Values are numbers (percentage) unless stated otherwise.**

| **Characteristics** | **Included sample** | **Excluded sample** |
| --- | --- | --- |
|  | (N=331,066) | (N=91,841) |
| **Socio-demographics and lifestyle factors:** |  |  |
| Men | 158,460 (48) | 42,218 (46) |
| Mean (SD) age (years) | 56.14 (8.08) | 56.20 (8.10) |
| Ethnicity |  |  |
| White | 317,813 (96) | 84,025 (93) |
| Non-white | 13,253 (4) | 6,719 (7) |
| Highest educational qualification |  |  |
| Other professional | 16,891 (5) | 4,620 (5) |
| NVQ/HND/HNC | 21,908 (7) | 6,096 (7) |
| CSEs | 17,649 (5) | 5,253 (6) |
| O levels/GCSEs | 71,338 (22) | 18,588 (20) |
| A levels/AS levels | 39,036 (12) | 10,214 (11) |
| College or University degree | 117,357 (35) | 30,971 (34) |
| Less than high school | 46,887 (14) | 16,099 (18) |
| Employment status |  |  |
| Employed or self-employed | 190,851 (58) | 50,737 (55) |
| Retired | 116,582 (35) | 31,510 (34) |
| Others | 23,633 (7) | 9,594 (10) |
| Household income |  |  |
| <£18,000 | 70,300 (21) | 25,617 (28) |
| £18,000 – £30,999 | 83,761 (25) | 23,770 (26) |
| £31,000 – £51,999 | 88,216 (27) | 22,209 (24) |
| £52,000 - £100,000 | 70,126 (21) | 16,004 (17) |
| ≥£100,000 | 18,663 (6) | 4,241 (5) |
| Healthy diet | 147,260 (44) | 37,952 (44) |
| Not frequent alcohol consumer | 259,604 (78) | 73,660 (80) |
| Not current smoker | 297,202 (90) | 79,969 (88) |
| Healthy lifestyle score |  |  |
| 0-1 | 62,371 (19) | 15,685 (19) |
| 2 | 157,680 (48) | 40,082 (48) |
| 3 | 111,015 (34) | 28,466 (34) |
| Number of social activities |  |  |
| None | 97,533 (29) | 29,308 (32) |
| One or more | 233,533 (71) | 61,809 (68) |
| **Clinical phenotypes** |  |  |
| Abdominal obesity^a^ |  |  |
| Obese | 162,259 (49) | 45,607 (50) |
| Mean (SD) Waist circumference (cm) | 90.27 (13.34) | 90.77 (13.85) |
| Mean (SD) Hip circumference (cm) | 103.31 (9.03) | 103.62 (9.63) |
| Frailty |  |  |
| Frail/pre-frail | 131,250 (40) | 30,612 (44) |
| Weight change compared with 1 year age |  |  |
| Weight-loss | 50,552 (15) | 13,342 (16) |
| Grip strength |  |  |
| Low grip strength | 42,408 (13) | 14,199 (16) |
| Gait speed |  |  |
| Slow gait speed | 22,920 (7) | 8,470 (9) |
| Exhaustion |  |  |
| More than half the days/nearly everyday | 38,490 (12) | 12,178 (15) |
| Physical activities |  |  |
| Low physical activity | 26,081 (8) | 10,299 (11) |
| Hearing difficulty |  |  |
| Deaf or having difficulty with hearing | 84,430 (26) | 19,360 (26) |
| Mean (SD) Leucocyte telomere length^b^ | 0.009 (1.00) | -0.004 (1.00) |
| Mean (SD) C-reactive protein (mg/L) | 2.51 (4.23) | 2.74 (4.63) |
| Comorbidities at baseline |  |  |
| Cancer | 19,756 (6) | 6,249 (7) |
| Cardiovascular disease | 46,703 (14) | 14,170 (15) |
| Diabetes | 15,928 (5) | 5,264 (6) |
| Hypertension | 94,283 (28) | 27,655 (30) |
| Psychiatric disorder | 38,738 (12) | 12,194 (13) |
| Respiratory disease | 71,676 (22) | 20,707 (23) |
| **Neighbourhood environment:** |  |  |
| Urbanicity |  |  |
| Tertile 1 (least urbanized) | 68,666 (21) | 13,604 (18) |
| Tertile 2-4 | 197,630 (60) | 46,220 (60) |
| Tertile 5 (most urbanized) | 64,770 (20) | 16,655 (22) |
| Townsend deprivation index |  |  |
| Tertile 1 (least deprived) | 69,809 (21) | 15,636 (17) |
| Tertiles 2-4 | 203,052 (61) | 52,167 (57) |
| Tertile 5 (most deprived) | 58,205 (18) | 23,491 (26) |

Values are numbers (percentage) unless stated otherwise.

^a^Obese is defined as waist–hip ratio (WHR) ≥0.90 cm for men or ≥0.85 cm for women. Non-obese is defined as WHR <0.90 cm for men or <0.85 cm for women.

^b^Leucocyte telomere length was Z-standardised, log transformed and adjusted for technical parameters.

**Table S6. Associations of biological age (measured as the adjusted residual of leucocyte telomere length, LTL) with risks of incident dementia and Alzheimer’s disease among UK Biobank participants (N=331,066).**

| **Biological age** |  | **Leucocyte telomere length (LTL) strata^b^** | | |  |
| --- | --- | --- | --- | --- | --- |
|  | Total | **Long**  (top 25^th^ percentile) | **Medium**  (25^th^ – 75^th^ percentile) | **Short**  (bottom 25^th^ percentile) | p-trend |
|  | N/Events | HR (95% CI) | HR (95% CI) | HR (95% CI) |  |
| **Incident dementia** | |  |  |  |  |
| Model^a^ | 331,066/3,313 | 1 – Ref | 1.17 (1.07-1.28) | 1.20 (1.09-1.32) | <0.001 |
| **Incident Alzheimer’s disease** | |  |  |  |  |
| Model^a^ | 331,066/1,423 | 1 - Ref | 1.23 (1.08-1.41) | 1.29 (1.11-1.50) | <0.001 |

^a^Model included age, sex, ethnicity, healthy lifestyle score, social activities, hearing difficulty, abdominal obesity, frailty index, urbanicity, neighbourhood deprivation, and individual-level socioeconomic status.

^b^Residual of LTL, adjusted for chronological age, C-reactive protein and co-morbidities was defined as biological age in the study.

CI=confidence interval; HR=hazard ratio; N=number of participants.

**Table S7. Joint associations of individual-level socioeconomic status (SES) and biological age (measured as leucocyte telomere length, LTL) with incident dementia and Alzheimer’s disease among UK Biobank participants (N=331,066).**

|  |  | **Individual-level SES** | | |
| --- | --- | --- | --- | --- |
|  |  | **High** | **Medium** | **Low** |
|  | N/Events | HR (95% CI) | HR (95% CI) | HR (95% CI) |
| **Incident dementia^a^** |  |  |  |  |
| 1 (top 25^th^ – long LTL^b^) | 82,518/702 | 1 – Ref | 1.73 (1.34-2.23) | 2.55 (1.98-3.28) |
| 2 | 165,723/1,703 | 1.25 (0.95-1.63) | 1.90 (1.50-2.42) | 3.09 (2.43-3.92) |
| 3 (bottom 25^th^ – short LTL) | 82,825/908 | 1.17 (0.86-1.59) | 1.90 (1.48-2.44) | 3.28 (2.57-4.20) |
| RERI | 0.57 (0.07-1.06) |  |  |  |
| **Incident Alzheimer’s disease^a^** |  |  |  |  |
| 1 (top 25^th^ – long LTL) | 82,518/292 | 1 - Ref | 1.64 (1.10-2.44) | 2.48 (1.68-3.67) |
| 2 | 165,723/736 | 1.23 (0.81-1.87) | 1.82 (1.25-2.64) | 3.31 (2.29-4.79) |
| 3 (bottom 25^th^ – short LTL) | 82,825/395 | 1.22 (0.75-1.97) | 1.94 (1.32-2.87) | 3.44 (2.35-5.04) |
| RERI | 0.79 (0.02-1.56) |  |  |  |

^a^Model included age, sex, ethnicity, healthy lifestyle score, social activities, hearing difficulty, abdominal obesity, frailty index, urbanicity and neighbourhood deprivation.

^b^Residual of LTL, adjusted for chronological age, C-reactive protein and co-morbidities was defined as biological age in the study.

RERI, relative excess risk due to interaction. Estimates for low SES and short LTL were labelled as RR11, low SES and long LTL as RR10, and high SES and short LTL as RR01. RERI was calculated as RR11 – RR10 – RR01 + 1.

CI=confidence interval; HR=hazard ratio; N=number of participants, LTL=leucocyte telomere length.

**Table S8. Associations between single individual-level socioeconomic factors and risks of incident dementia and Alzheimer’s disease among UK Biobank participants (N=331,066).**

|  | **Incident dementia^a^** | |  | **Incident Alzheimer’s disease^a^** | |
| --- | --- | --- | --- | --- | --- |
|  | N/Events | HR (95% CI) |  | N/Events | HR (95% CI) |
| **Education^b^** |  |  |  |  |  |
| High | 117,357/786 | 1 - Ref |  | 117,357/329 | 1 - Ref |
| Medium | 166,822/1,526 | 1.07 (0.98-1.17) |  | 166,822/641 | 1.08 (0.94-1.23) |
| Low | 46,887/1,001 | 1.21 (1.09-1.34) |  | 46,887/453 | 1.32 (1.13-1.54) |
| **Employment status** |  |  |  |  |  |
| Employed or self-employed | 190,851/730 | 1 - Ref |  | 190,851/307 | 1 - Ref |
| Retired | 116,582/2,366 | 1.59 (1.45-1.75) |  | 116,582/1,030 | 1.51 (1.31-1.75) |
| Others | 23,633/217 | 1.61 (1.37-1.88) |  | 23,633/86 | 1.55 (1.21-1.98) |
| **Household income** |  |  |  |  |  |
| >£52,000 | 88,789/277 | 1 - Ref |  | 88,789/118 | 1 – Ref |
| £18,000-£51,999 | 171,977/1,561 | 1.49 (1.31-1.71) |  | 171,977/661 | 1.42 (1.16-1.73) |
| <£18,000 | 70,300/1,475 | 2.08 (1.80-2.40) |  | 70,300 | 1.99 (1.59-2.48) |

^a^Model included age, sex, ethnicity, healthy lifestyle score, social activities, hearing difficulty, abdominal obesity, frailty index, urbanicity, neighbourhood deprivation and adjusted residual leucocyte telomere length. When the association between a single socioeconomic factor and incident dementia or Alzheimer’s disease was examined, the model was adjusted for the other two socioeconomic factors.

^b^Education is categorized as high (i.e., college or university degree), medium (i.e., A levels/AS levels, O levels/GCSEs, CSEs, NVQ or HND or HNC, other professional qualifications) and low levels of education (i.e. none of the above).

CI=confidence interval; HR=hazard ratio; N=number of participants.

**Table S9. Associations between single individual-level socioeconomic factors and risks of incident dementia and Alzheimer’s disease stratified by biological age (measured as leucocyte telomere length, LTL) among UK Biobank participants (N=331,066).**

|  | **Incident dementia^a^** | | |  | **Incident Alzheimer’s disease^a^** | | |
| --- | --- | --- | --- | --- | --- | --- | --- |
|  | Leucocyte telomere length (LTL)^b^ | | |  | Leucocyte telomere length (LTL)^b^ | | |
|  | Long  (top 25^th^) | Medium  (25^th^ – 75^th^) | Short  (bottom 25^th^) |  | Long  (top 25th) | Medium  (25^th^ – 75^th^) | Short  (bottom 25^th^) |
|  | HR (95% CI) | HR (95% CI) | HR (95% CI) |  | HR (95% CI) | HR (95% CI) | HR (95% CI) |
| **Education^c^** |  |  |  |  |  |  |  |
| High | 1 - Ref | 1 - Ref | 1 - Ref |  | 1 - Ref | 1 - Ref | 1 - Ref |
| Medium | 1.01 (0.84-1.22) | 1.05 (0.93-1.19) | 1.16 (0.97-1.38) |  | 0.99 (0.74-1.32) | 1.06 (0.88-1.29) | 1.19 (0.91-1.57) |
| Low | 1.01 (0.81-1.26) | 1.20 (1.04-1.39) | 1.41 (1.16-1.73) |  | 0.97 (0.69-1.38) | 1.31 (1.06-1.64) | 1.65 (1.22-2.24) |
| **Employment status** |  |  |  |  |  |  |  |
| Employed or self-employed | 1 – Ref | 1 – Ref | 1 – Ref |  | 1 - Ref | 1 – Ref | 1 – Ref |
| Retired | 1.79 (1.45-2.21) | 1.47 (1.29-1.68) | 1.70 (1.41-2.04) |  | 1.60 (1.16-2.20) | 1.42 (1.17-1.73) | 1.62 (1.23-2.13) |
| Others^d^ | 1.72 (1.22-2.44) | 1.33 (1.06-1.68) | 2.05 (1.55-2.70) |  | 1.81 (1.07-3.08) | 1.39 (0.98-1.97) | 1.70 (1.08-2.69) |
| **Household income** |  |  |  |  |  |  |  |
| >£52,000 | 1 – Ref | 1 – Ref | 1 – Ref |  | 1 – Ref | 1 – Ref | 1 – Ref |
| £18,000-£51,999 | 1.46 (1.10-1.93) | 1.48 (1.23-1.78) | 1.55 (1.19-2.01) |  | 1.22 (0.81-1.86) | 1.43 (1.07-1.90) | 1.57 (1.05-2.34) |
| <£18,000 | 1.94 (1.43-2.64) | 2.15 (1.76-2.62) | 2.07 (1.55-2.75) |  | 1.60 (1.01-2.53) | 2.22 (1.63-3.02) | 1.92 (1.24-2.96) |

^a^Model included age, sex, ethnicity, healthy lifestyle score, social activities, hearing difficulty, abdominal obesity, frailty index, urbanicity, and neighbourhood deprivation. When the association between a single socioeconomic factor and incident dementia or Alzheimer’s disease was examined, the model was adjusted for the other two socioeconomic factors.

^b^Residual of LTL, adjusted for chronological age, C-reactive protein and co-morbidities was defined as biological age in the study.

^c^Education is categorized as high (i.e., college or university degree), medium (i.e., A levels/AS levels, O levels/GCSEs, CSEs, NVQ or HND or HNC, other professional qualifications) and low levels of education (i.e. none of the above).

^d^Others included looking after home, being unable to work due to sickness or disability, unemployed, doing unpaid or voluntary work or student.

CI=confidence interval; HR=hazard ratio; N=number of participants.

**Table S10. Joint associations of single individual-level socioeconomic factors and telomere length with incident dementia among UK Biobank participants (N=331,066).**

|  | **Incident dementia^a^** | | |  | **Incident Alzheimer’s disease^a^** | | |
| --- | --- | --- | --- | --- | --- | --- | --- |
|  | Leucocyte telomere length (LTL)^b^ | | |  | Leucocyte telomere length (LTL)^b^ | | |
|  | Long  (top 25^th^) | Medium  (25^th^ – 75^th^) | Short  (bottom 25^th^) |  | Long  (top 25^th^) | Medium  (25^th^ – 75^th^) | Short  (bottom 25^th^) |
|  | HR (95% CI) | HR (95% CI) | HR (95% CI) |  | HR (95% CI) | HR (95% CI) | HR (95% CI) |
| **Education^c^** |  |  |  |  |  |  |  |
| High | 1 - Ref | 1.02 (0.85-1.22) | 1.03 (0.83-1.26) |  | 1 - Ref | 1.00 (0.75-1.31) | 1.01 (0.74-1.40) |
| Medium | 1.10 (0.92-1.30) | 1.17 (1.00-1.37) | 1.33 (1.12-1.58) |  | 1.09 (0.83-1.42) | 1.18 (0.92-1.51) | 1.50 (1.15-1.95) |
| Low | 1.05 (0.86-1.28) | 1.19 (1.00-1.41) | 1.44 (1.20-1.74) |  | 1.09 (0.80-1.48) | 1.24 (0.95-1.62) | 1.59 (1.20-2.11) |
| RERI | 0.36 (0.08-0.65) |  |  |  | 0.52 (0.05-0.99) |  |  |
| **Employment status** |  |  |  |  |  |  |  |
| Employed or self-employed |  |  |  |  |  |  |  |
| Retired | 1 - Ref | 1.67 (1.38-2.01) | 1.66 (1.19-2.34) |  | 1 - Ref | 1.46 (1.10-1.95) | 1.72 (1.02-2.88) |
| Others^d^ | 1.26 (1.04-1.52) | 1.93 (1.62-2.31) | 1.68 (1.30-2.18) |  | 1.22 (0.91-1.63) | 1.84 (1.41-2.41) | 1.74 (1.17-2.59) |
| RERI | 1.18 (0.95-1.47) | 1.96 (1.63-2.36) | 2.48 (1.87-3.28) |  | 1.24 (0.89-1.72) | 1.92 (1.45-2.54) | 2.05 (1.29-3.25) |
| **Household income** | 0.64 (-0.15-1.43) |  |  |  | 0.06 (-1.09-1.20) |  |  |
| >£52,000 | 1 - Ref | 1.46 (1.11-1.91) | 1.94 (1.47-2.56) |  | 1 - Ref | 1.23 (0.82-1.84) | 1.63 (1.08-2.48) |
| £18,000-£51,999 | 1.13 (0.84-1.51) | 1.66 (1.28-2.15) | 2.35 (1.80-3.07) |  | 1.01 (0.65-1.58) | 1.44 (0.98-2.12) | 2.20 (1.49-3.26) |
| <£18,000 | 1.10 (0.78-1.54) | 1.72 (1.31-2.24) | 2.41 (1.83-3.17) |  | 1.06 (0.64-1.77) | 1.67 (1.12-2.47) | 2.08 (1.38-3.12) |
| RERI | 0.38 (-0.08-0.84) |  |  |  | 0.47 (-0.21-1.14) |  |  |

^a^Model included age, sex, ethnicity, healthy lifestyle score, social activities, hearing difficulty, abdominal obesity, frailty index, urbanicity, and neighbourhood deprivation. When the association between a single socioeconomic factor and incident dementia or Alzheimer’s disease was examined, the model was adjusted for the other two socioeconomic factors.

^b^Residual of LTL, adjusted for chronological age, C-reactive protein and co-morbidities was defined as biological age in the study.

^c^Education is categorized as high (i.e., college or university degree), medium (i.e., A levels/AS levels, O levels/GCSEs, CSEs, NVQ or HND or HNC, other professional qualifications) and low levels of education (i.e. none of the above).

^d^Others included looking after home, being unable to work due to sickness or disability, unemployed, doing unpaid or voluntary work or student.

RERI, relative excess risk due to interaction. Estimates for low SES and short LTL were labelled as RR11, low SES and long LTL as RR10, and high SES and short LTL as RR01. RERI was calculated as RR11 – RR10 – RR01 + 1.

CI=confidence interval; HR=hazard ratio; N=number of participants.

**Table S11. Sensitivity analysis for combined cases of incident dementia diagnosis and mortality as outcome.**

|  | **Individual-level SES^d^** | | | |
| --- | --- | --- | --- | --- |
|  |  | High | Medium | Low |
|  | N/Events | HR (95% CI) | HR (95% CI) | HR (95% CI) |
| **Primary analysis^ae^** |  |  |  |  |
| Fully-adjusted model | 331,066/3,485 | 1 - Ref | 1.62 (1.44-1.82) | 2.57 (2.28-2.90) |
|  |  |  |  |  |
| **Stratified analysis^be^** |  |  |  |  |
| 1 (top 25^th^ – long LTL) | 82,518/736 | 1 - Ref | 1.69 (1.32-2.18) | 2.44 (1.89-3.15) |
| 2 | 165,723/1,792 | 1 - Ref | 1.53 (1.30-1.80) | 2.44 (2.07-2.87) |
| 3 (bottom 25^th^ – short LTL) | 82,825/957 | 1 - Ref | 1.75 (1.38-2.22) | 2.98 (2.34-3.79) |
|  |  |  |  |  |
| **Joint associations^ce^** |  |  |  |  |
| 1 (top 25^th^ – long LTL) | 82,518/736 | 1 - Ref | 1.69 (1.32-2.16) | 2.48 (1.94-3.17) |
| 2 | 165,723/1,792 | 1.22 (0.94-1.58) | 1.89 (1.50-2.38) | 3.00 (2.38-3.78) |
| 3 (bottom 25^th^ – short LTL) | 82,825/957 | 1.11 (0.82-1.50) | 1.89 (1.48-2.41) | 3.21 (2.53-4.08) |
| RERI | 0.62 (0.16-1.09) |  |  |  |

^a^Associations of individual-level SES with risks of incident dementia and mortality.

^b^Associations between individual-level SES and risks of incident dementia and mortality stratified by adjusted residual telomere length.

^c^Joint associations of individual-level SES and telomere length with incident dementia and mortality.

^d^Model included age, sex, ethnicity, healthy lifestyle score, social activities, hearing difficulty, abdominal obesity, frailty index, urbanicity, neighbourhood deprivation, and adjusted residual leucocyte telomere length (in primary analysis).

RERI, relative excess risk due to interaction. Estimates for low SES and short LTL were labelled as RR11, low SES and long LTL as RR10, and high SES and short LTL as RR01. RERI was calculated as RR11 – RR10 – RR01 + 1.

^e^Residual of LTL, adjusted for chronological age, C-reactive protein and co-morbidities was defined as biological age in the study. CI=confidence interval; HR=hazard ratio; N=number of participants; LTL=leucocyte telomere length.

**Table S12. Sensitivity analysis rerunning latent class analysis based on multiple individual socioeconomic factors (education, employment status and household income) and neighborhood deprivation measured by Townsend index of deprivation.**

|  | **Incident dementia^d^** | | |  | **Incident Alzheimer’s disease^d^** | | |
| --- | --- | --- | --- | --- | --- | --- | --- |
|  | Individual-level SES | | |  | Individual-level SES | | |
|  | High | Medium | Low |  | High | Medium | Low |
|  | HR (95% CI) | HR (95% CI) | HR (95% CI) |  | HR (95% CI) | HR (95% CI) | HR (95% CI) |
| N/Events | 159,413/604 | 103,179/1,231 | 68,474/1,478 |  | 159,413/248 | 103,179/527 | 68,474/648 |
| **Primary analysis^ae^** |  |  |  |  |  |  |  |
| Fully-adjusted model | 1 - Ref | 1.61 (1.46-1.78) | 2.46 (2.22-2.72) |  | 1 - Ref | 1.60 (1.37-1.87) | 2.48 (2.13-2.90) |
|  |  |  |  |  |  |  |  |
| **Stratified analysis^be^** |  |  |  |  |  |  |  |
| 1 (top 25^th^ – long LTL) | 1 - Ref | 1.48 (1.20-1.83) | 2.12 (1.72-2.62) |  | 1 - Ref | 1.59 (1.14-2.22) | 2.15 (1.53-3.01) |
| 2 | 1 - Ref | 1.58 (1.38-1.82) | 2.46 (2.14-2.83) |  | 1 - Ref | 1.50 (1.21-1.87) | 2.56 (2.07-3.17) |
| 3 (bottom 25^th^ – short LTL) | 1 - Ref | 1.79 (1.47-2.18) | 2.75 (2.26-3.36) |  | 1 - Ref | 1.78 (1.32-2.40) | 2.61 (1.93-3.52) |
|  |  |  |  |  |  |  |  |
| **Joint associations^ce^** |  |  |  |  |  |  |  |
| 1 (top 25^th^ – long LTL) | 1 - Ref | 1.49 (1.22-1.83) | 2.18 (1.78-2.67) |  | 1 - Ref | 1.61 (1.16-2.23) | 2.28 (1.65-3.15) |
| 2 | 1.07 (0.88-1.31) | 1.73 (1.44-2.07) | 2.67 (2.23-3.20) |  | 1.19 (0.86-1.63) | 1.82 (1.35-2.44) | 3.07 (2.30-4.11) |
| 3 (bottom 25^th^ – short LTL) | 1.04 (0.83-1.31) | 1.81 (1.48-2.20) | 2.75 (2.27-3.34) |  | 1.23 (0.85-1.76) | 2.10 (1.53-2.86) | 2.99 (2.19-4.07) |
| RERI | 0.52 (0.10-0.94) |  |  |  | 0.49 (-0.22-1.20) |  |  |

^a^Associations of individual-level SES with risks of incident dementia and Alzheimer’s disease.

^b^Associations between individual-level SES and risks of incident dementia and Alzheimer’s disease stratified by adjusted residual telomere length.

^c^Joint associations of individual-level SES and telomere length with incident dementia and Alzheimer’s disease.

^d^Model included age, sex, ethnicity, healthy lifestyle score, social activities, hearing difficulty, abdominal obesity, frailty index, urbanicity, residual adjusted leucocyte telomere length (in primary analysis).

^e^Residual of LTL, adjusted for chronological age, C-reactive protein and co-morbidities was defined as biological age in the study. RERI, relative excess risk due to interaction. Estimates for low SES and short LTL were labelled as RR11, low SES and long LTL as RR10, and high SES and short LTL as RR01. RERI was calculated as RR11 – RR10 – RR01 + 1.

CI=confidence interval; HR=hazard ratio; N=number of participants; LTL=leucocyte telomere length; SES, socioeconomic status.

**Table S13. Sensitivity analysis with additional adjustment for number of household members.**

|  | **Incident dementia^d^** | | |  | **Incident Alzheimer’s disease^d^** | | |
| --- | --- | --- | --- | --- | --- | --- | --- |
|  | Individual-level SES | | |  | Individual-level SES | | |
|  | High | Medium | Low |  | High | Medium | Low |
|  | HR (95% CI) | HR (95% CI) | HR (95% CI) |  | HR (95% CI) | HR (95% CI) | HR (95% CI) |
| **Primary analysis^ae^** |  |  |  |  |  |  |  |
| Fully-adjusted model | 1 - Ref | 1.55 (1.37-1.75) | 2.45 (2.16-2.78) |  | 1 - Ref | 1.51 (1.25-1.83) | 2.63 (2.17-3.20) |
|  |  |  |  |  |  |  |  |
| **Stratified analysis^be^** |  |  |  |  |  |  |  |
| 1 (top 25^th^ – long LTL) | 1 - Ref | 1.71 (1.31-2.22) | 2.46 (1.88-3.23) |  | 1 - Ref | 1.65 (1.09-2.48) | 2.39 (1.57-3.64) |
| 2 | 1 - Ref | 1.45 (1.23-1.72) | 2.30 (1.94-2.73) |  | 1 - Ref | 1.42 (1.09-1.85) | 2.58 (1.98-3.36) |
| 3 (bottom 25^th^ – short LTL) | 1 - Ref | 1.63 (1.28-2.08) | 2.77 (2.16-3.54) |  | 1 - Ref | 1.60 (1.10-2.33) | 2.98 (2.04-4.35) |
|  |  |  |  |  |  |  |  |
| **Joint associations^ce^** |  |  |  |  |  |  |  |
| 1 (top 25^th^ – long LTL) | 1 - Ref | 1.68 (1.30-2.18) | 2.43 (1.88-3.14) |  | 1 - Ref | 1.61 (1.08-2.40) | 2.45 (1.65-3.63) |
| 2 | 1.25 (0.95-1.63) | 1.85 (1.46-2.36) | 2.93 (2.31-3.73) |  | 1.23 (0.81-1.87) | 1.79 (1.23-2.60) | 3.26 (2.25-4.73) |
| 3 (bottom 25^th^ – short LTL) | 1.17 (0.86-1.59) | 1.84 (1.43-2.37) | 3.12 (2.44-4.00) |  | 1.22 (0.75-1.97) | 1.90 (1.28-2.81) | 3.36 (2.29-4.93) |
| RERI | 0.53 (0.05-1.01) |  |  |  | 0.76 (-0.02-1.53) |  |  |

^a^Associations of individual-level SES with risks of incident dementia and Alzheimer’s disease.

^b^Associations between individual-level SES and risks of incident dementia and Alzheimer’s disease stratified by adjusted residual telomere length.

^c^Joint associations of individual-level SES and telomere length with incident dementia and Alzheimer’s disease.

^d^Model included age, sex, ethnicity, healthy lifestyle score, social activities, hearing difficulty, abdominal obesity, frailty index, urbanicity, neighbourhood deprivation, adjusted residual leucocyte telomere length (in primary analysis), and number of household members.

^e^Residual of LTL, adjusted for chronological age, C-reactive protein and co-morbidities was defined as biological age in the study. RERI, relative excess risk due to interaction. Estimates for low SES and short LTL were labelled as RR11, low SES and long LTL as RR10, and high SES and short LTL as RR01. RERI was calculated as RR11 – RR10 – RR01 + 1.

CI=confidence interval; HR=hazard ratio; N=number of participants; LTL=leucocyte telomere length; SES, socioeconomic status.

**Table S14. Sensitivity analysis with additional adjustment for comorbidities as covariates.**

|  | **Incident dementia^d^** | | | |  | **Incident Alzheimer’s disease^d^** | | | |
| --- | --- | --- | --- | --- | --- | --- | --- | --- | --- |
|  | Individual-level SES | | | |  | Individual-level SES | | | |
|  | High | Medium | | Low |  | High | Medium | | Low |
|  | HR (95% CI) | HR (95% CI) | | HR (95% CI) |  | HR (95% CI) | HR (95% CI) | | HR (95% CI) |
| **Primary analysis^ae^** |  |  | |  |  |  |  | |  |
| Fully-adjusted model | 1 - Ref | 1.55 (1.37-1.75) | | 2.39 (2.11-2.70) |  | 1 - Ref | 1.52 (1.26-1.84) | | 2.59 (2.14-3.13) |
|  |  |  | |  |  |  |  | |  |
| **Stratified analysis^be^** |  |  | |  |  |  |  | |  |
| 1 (top 25^th^ – long LTL) | 1 - Ref | 1.69 (1.30-2.19) | | 2.30 (1.76-2.99) |  | 1 - Ref | 1.58 (1.05-2.36) | | 2.17 (1.44-3.27) |
| 2 | 1 - Ref | 1.45 (1.23-1.71) | | 2.26 (1.91-2.67) |  | 1 - Ref | 1.43 (1.10-1.85) | | 2.55 (1.97-3.31) |
| 3 (bottom 25^th^ – short LTL) | 1 - Ref | 1.65 (1.30-2.10) | | 2.74 (2.15-3.50) |  | 1 - Ref | 1.68 (1.16-2.43) | | 3.07 (2.11-4.46) |
|  |  |  | |  |  |  |  | |  |
| **Joint associations^ce^** |  |  | |  |  |  |  | |  |
| 1 (top 25^th^ – long LTL) | 1 - Ref | 1.69 (1.31-2.18) | | 2.37 (1.84-3.05) |  | 1 - Ref | 1.62 (1.09-2.41) | | 2.40 (1.62-3.56) |
| 2 | 1.25 (0.96-1.63) | 1.85 (1.46-2.35) | | 2.87 (2.26-3.65) |  | 1.24 (0.81-1.88) | 1.80 (1.24-2.61) | | 3.21 (2.22-4.65) |
| 3 (bottom 25^th^ – short LTL) | 1.17 (0.85-1.59) | 1.85 (1.44-2.38) | | 3.02 (2.36-3.86) |  | 1.21 (0.75-1.96) | 1.92 (1.30-2.84) | | 3.31 (2.26-4.85) |
| RERI | 0.50 (0.03-0.98) | |  | |  | 0.74 (-0.01-1.49) | |  | |

^a^Associations of individual-level SES with risks of incident dementia and Alzheimer’s disease

^b^Associations between individual-level SES and risks of incident dementia and Alzheimer’s disease stratified by adjusted residual telomere length.

^c^Joint associations of individual-level SES and telomere length with incident dementia and Alzheimer’s disease.

^d^Model included age, sex, ethnicity, healthy lifestyle score, social activities, hearing difficulty, abdominal obesity, frailty index, urbanicity, neighbourhood deprivation, adjusted residual leucocyte telomere length (in primary analysis), and forty six chronic comorbidities (coded as cardiovascular diseases, cancer, diabetes, hypertension, psychiatric disorders and respiratory diseases).

RERI, relative excess risk due to interaction. Estimates for low SES and short LTL were labelled as RR11, low SES and long LTL as RR10, and high SES and short LTL as RR01. RERI was calculated as RR11 – RR10 – RR01 + 1.

^e^Leucocyte telomere length was Z-standardised, log transformed and adjusted for technical parameters, chronological age, and C-reactive protein.

CI=confidence interval; HR=hazard ratio; N=number of participants; LTL=leucocyte telomere length.

**Table S15. Sensitivity analysis examining associations of occupation categories with incident and Alzheimer’s disease for the employed sub-group (N=190,426).**

|  | **Occupation categories** | | | | |
| --- | --- | --- | --- | --- | --- |
|  | Other occupations^c^ | Associate Professional and Technical Occupations | Professional Occupations | Managers and Senior Officials | Administrative and Secretariat Occupations |
|  | HR (95% CI) | HR (95% CI) | HR (95% CI) | HR (95% CI) | HR (95% CI) |
| **Incident dementia** |  |  |  |  |  |
| Fully-adjusted model^a^ | 1 - Ref | 1.17 (0.93-1.47) | 0.84 (0.65-1.08) | 0.95 (0.75-1.22) | 0.98 (0.77-1.25) |
|  |  |  |  |  |  |
| **Incident Alzheimer’s disease** |  |  |  |  |  |
| Fully-adjusted model^a^ | 1 - Ref | 1.24 (0.87-1.75) | 0.99 (0.67-1.44) | 1.06 (0.72-1.56) | 0.83 (0.56-1.22) |

^a^Associations of individual-level SES with risks of incident dementia and Alzheimer’s disease.

^b^Model included age, sex, education, household income, ethnicity, healthy lifestyle score, social activities, hearing difficulty, abdominal obesity, frailty index, urbanicity, neighbourhood deprivation, and adjusted residual leucocyte telomere length (in primary analysis).

^c^Skilled trades occupations/personal service occupations/sales and customer service occupations/process, plant and machine operatives/elementary occupations.

CI=confidence interval; HR=hazard ratio; N=number of participants; LTL=leucocyte telomere length.

**Table S16. Primary and stratified analysis and joint associations of individual-level SES and LTL with incident dementia by age in the study.**

|  | **<65 years** | | |  | **≥65 years** | | |
| --- | --- | --- | --- | --- | --- | --- | --- |
|  | Individual-level SES^d^  (N=113,011/233) | | |  | Individual-level SES^d^  (N=218,055/3,080) | | |
|  | High | Medium | Low |  | High | Medium | Low |
|  | HR (95% CI) | HR (95% CI) | HR (95% CI) |  | HR (95% CI) | HR (95% CI) | HR (95% CI) |
| **Primary analysis^ae^** |  |  |  |  |  |  |  |
| Fully-adjusted model | 1 - Ref | 1.57 (1.11-2.22) | 5.14 (3.62-7.30) |  | 1 - Ref | 2.37 (2.08-2.69) | 4.58 (4.03-5.21) |
|  |  |  |  |  |  |  |  |
| **Stratified analysis^be^** |  |  |  |  |  |  |  |
| 1 (top 25^th^ – long LTL) | 1 - Ref | 1.42 (0.72-2.80) | 4.18 (2.07-8.45) |  | 1 - Ref | 2.75 (2.07-3.66) | 4.66 (3.50-6.20) |
| 2 | 1 - Ref | 1.47 (0.91-2.38) | 4.46 (2.73-7.30) |  | 1 - Ref | 2.24 (1.88-2.67) | 4.41 (3.70-5.25) |
| 3 (bottom 25^th^ – short LTL) | 1 - Ref | 2.04 (0.98-4.25) | 8.27 (3.98-17.17) |  | 1 - Ref | 2.34 (1.81-3.01) | 4.86 (3.78-6.25) |
|  |  |  |  |  |  |  |  |
| **Joint associations^ce^** |  |  |  |  |  |  |  |
| 1 (top 25^th^ – long LTL) | 1 - Ref | 1.35 (0.69-2.66) | 3.88 (2.00-7.51) |  | 1 - Ref | 2.79 (2.10-3.71) | 4.90 (3.70-6.49) |
| 2 | 0.88 (0.49-1.59) | 1.38 (0.78-2.45) | 4.56 (2.59-8.04) |  | 1.36 (1.01-1.84) | 3.05 (2.33-4.00) | 5.92 (4.53-7.73) |
| 3 (bottom 25^th^ – short LTL) | 0.68 (0.32-1.42) | 1.27 (0.66-2.46) | 4.57 (2.46-8.50) |  | 1.32 (0.94-1.87) | 3.06 (2.31-4.05) | 6.37 (4.84-8.38) |

^a^Associations of individual-level SES with risks of incident dementia.

^b^Associations between individual-level SES and risks of incident dementia stratified by adjusted residual leucocyte telomere length.

^c^Joint associations of individual-level SES and telomere length with incident dementia.

^d^Model included sex, ethnicity, healthy lifestyle score, social activities, hearing difficulty, abdominal obesity, frailty index, urbanicity, neighbourhood deprivation, and adjusted residual leucocyte telomere length (in primary analysis).

^e^Residual of LTL, adjusted for chronological age, C-reactive protein and co-morbidities was defined as biological age in the study. CI=confidence interval; HR=hazard ratio; N=number of participants; LTL=leucocyte telomere length.

**Table S17. Primary and stratified analysis and joint associations of individual-level SES and LTL with incident Alzheimer’s disease by age in the study.**

|  | **<65 years** | | |  | **≥65 years** | | |
| --- | --- | --- | --- | --- | --- | --- | --- |
|  | Individual-level SES^d^  (N=112,948/83) | | |  | Individual-level SES^d^  (N=218,118/1,340) | | |
|  | High | Medium | Low |  | High | Medium | Low |
|  | HR (95% CI) | HR (95% CI) | HR (95% CI) |  | HR (95% CI) | HR (95% CI) | HR (95% CI) |
| **Primary analysis^ae^** |  |  |  |  |  |  |  |
| Fully-adjusted model | 1 - Ref | 1.35 (0.76-2.40) | 5.49 (3.12-9.67) |  | 1 - Ref | 2.40 (1.97-2.94) | 5.03 (4.13-6.14) |
|  |  |  |  |  |  |  |  |
| **Stratified analysis^be^** |  |  |  |  |  |  |  |
| 1 (top 25^th^ – long LTL) | 1 - Ref | 1.10 (0.38-3.20) | 4.12 (1.43-11.89) |  | 1 - Ref | 2.75 (1.75-4.31) | 4.59 (2.92-7.20) |
| 2 | 1 - Ref | 0.85 (0.34-2.13) | 4.77 (2.10-10.86) |  | 1 - Ref | 2.29 (1.74-3.01) | 4.99 (3.81-6.53) |
| 3 (bottom 25^th^ – short LTL) | 1 - Ref | 3.07 (1.03-9.09) | 8.70 (2.64-28.72) |  | 1 - Ref | 2.40 (1.62-3.55) | 5.50 (3.74-8.10) |
|  |  |  |  |  |  |  |  |
| **Joint associations^ce^** |  |  |  |  |  |  |  |
| 1 (top 25^th^ – long LTL) | 1 - Ref | 1.09 (0.38-3.14) | 3.94 (1.44-10.76) |  | 1 - Ref | 2.88 (1.84-4.52) | 5.22 (3.35-8.14) |
| 2 | 0.67 (0.27-1.68) | 0.66 (0.25-1.76) | 4.46 (1.90-10.48) |  | 1.43 (0.89-2.30) | 3.26 (2.12-4.99) | 6.99 (4.58-10.68) |
| 3 (bottom 25^th^ – short LTL) | 0.61 (0.20-1.87) | 1.57 (0.62-4.01) | 3.45 (1.26-9.42) |  | 1.43 (0.84-2.46) | 3.34 (2.14-5.20) | 7.42 (4.82-11.44) |

^a^Associations of individual-level SES with risks of Alzheimer’s disease.

^b^Associations between individual-level SES and risks of Alzheimer’s disease stratified by adjusted residual leucocyte telomere length.

^c^Joint associations of individual-level SES and telomere length with Alzheimer’s disease.

^d^Model included sex, ethnicity, healthy lifestyle score, social activities, hearing difficulty, abdominal obesity, frailty index, urbanicity, neighbourhood deprivation, and adjusted residual leucocyte telomere length (in primary analysis).

^e^Residual of LTL, adjusted for chronological age, C-reactive protein and co-morbidities was defined as biological age in the study. CI=confidence interval; HR=hazard ratio; N=number of participants; LTL=leucocyte telomere length.

**Table S18. Primary and stratified analysis and joint associations of individual-level SES and LTL with incident dementia and Alzheimer’s disease with data imputed for missingness across covariates (N=422,907).**

|  | **Incident dementia^d^** | | |  | **Incident Alzheimer’s disease^d^** | | |
| --- | --- | --- | --- | --- | --- | --- | --- |
|  | Individual-level SES | | |  | Individual-level SES | | |
|  | High | Medium | Low |  | High | Medium | Low |
|  | HR (95% CI) | HR (95% CI) | HR (95% CI) |  | HR (95% CI) | HR (95% CI) | HR (95% CI) |
| **Primary analysis^ae^** |  |  |  |  |  |  |  |
| Fully-adjusted model | 1 - Ref | 1.55 (1.39-1.73) | 2.55 (2.29-2.84) |  | 1 - Ref | 1.53 (1.29-1.80) | 2.53 (2.15-2.99) |
|  |  |  |  |  |  |  |  |
| **Stratified analysis^bef^** |  |  |  |  |  |  |  |
| 1 (top 25^th^ – long LTL) | 1 - Ref | 1.55 (1.23-1.95) | 2.33 (1.84-2.94) |  | 1 - Ref | 1.53 (1.07-2.17) | 2.13 (1.49-3.04) |
| 2 | 1 - Ref | 1.48 (1.27-1.72) | 2.42 (2.09-2.82) |  | 1 - Ref | 1.47 (1.16-1.87) | 2.56 (2.03-3.24) |
| 3 (bottom 25^th^ – short LTL) | 1 - Ref | 1.71 (1.37-2.13) | 3.04 (2.44-3.80) |  | 1 - Ref | 1.64 (1.17-2.28) | 2.85 (2.04-3.96) |
|  |  |  |  |  |  |  |  |
| **Joint associations^ce^** |  |  |  |  |  |  |  |
| 1 (top 25^th^ – long LTL) | 1 - Ref | 1.55 (1.24-1.95) | 2.39 (1.91-2.99) |  | 1 - Ref | 1.55 (1.10-2.19) | 2.28 (1.62-3.21) |
| 2 | 1.15 (0.90-1.46) | 1.73 (1.40-2.14) | 2.85 (2.31-3.52) |  | 1.12 (0.77-1.62) | 1.67 (1.20-2.32) | 2.93 (2.11-4.05) |
| 3 (bottom 25^th^ – short LTL) | 1.09 (0.82-1.44) | 1.80 (1.44-2.25) | 3.11 (2.50-3.87) |  | 1.20 (0.78-1.84) | 1.89 (1.34-2.66) | 3.11 (2.23-4.36) |

^a^Associations of individual-level SES with risks of incident dementia and Alzheimer’s disease.

^b^Associations between individual-level SES and risks of incident dementia and Alzheimer’s disease stratified by adjusted residual leucocyte telomere length.

^c^Joint associations of individual-level SES and telomere length with incident dementia and Alzheimer’s disease.

^d^Model included age, sex, ethnicity, healthy lifestyle score, social activities, hearing difficulty, abdominal obesity, frailty index, urbanicity, neighbourhood deprivation, and adjusted residual leucocyte telomere length (in primary analysis).

^e^Residual of LTL, adjusted for chronological age, C-reactive protein and co-morbidities was defined as biological age in the study.

^f^Total number varies among imputations; 105,123 to 105,538 for long LTL; 211,395 to 211,720 for medium LTL; and 105,863 to 106,208 for short LTL.

The imputation model imputed missingness across covariates in the fully-adjusted model and all covariates, and exposure and outcome variables were included. A total of 20 imputation sets were created.

CI=confidence interval; HR=hazard ratio; N=number of participants; LTL=leucocyte telomere length; SES, socioeconomic status.

191 excluded due to study withdrawal during follow-up

377 excluded due to dementia/Alzheimer’s disease diagnosis at baseline or during landmark period

502,650 baseline participants of UK Biobank

502,082 participants over the study period

79,175 excluded due to missingness on one or more individual-level SES (exposure)

10,115 for education

1,255 for employment status

67,805 for household income

422,907 participants with complete data on both exposure and confounders

91,841 excluded due to missingness on one or more confounder(s)

9,196 for other socio-demographics and

lifestyle

69,871 for clinical phenotypes

12,774 for neighbourhood built

environment

331,066 participants

(analytic sample)

**Figure S1. Flowchart for participant selection.**
